# Analyzing Neuroimaging Data Through Recurrent Deep Learning Models

**DOI:** 10.3389/fnins.2019.01321

**Published:** 2019-12-10

**Authors:** Armin W. Thomas, Hauke R. Heekeren, Klaus-Robert Müller, Wojciech Samek

**Affiliations:** ^1^Machine Learning Group, Technische Universität Berlin, Berlin, Germany; ^2^Center for Cognitive Neuroscience Berlin, Freie Universität Berlin, Berlin, Germany; ^3^Max Planck School of Cognition, Leipzig, Germany; ^4^Department of Education and Psychology, Freie Universität Berlin, Berlin, Germany; ^5^Department of Brain and Cognitive Engineering, Korea University, Seoul, South Korea; ^6^Max Planck Institute for Informatics, Saarbrücken, Germany; ^7^Machine Learning Group, Fraunhofer Heinrich Hertz Institute, Berlin, Germany

**Keywords:** decoding, neuroimaging, fMRI, whole-brain, deep learning, recurrent, interpretability

## Abstract

The application of deep learning (DL) models to neuroimaging data poses several challenges, due to the high dimensionality, low sample size, and complex temporo-spatial dependency structure of these data. Even further, DL models often act as *black boxes*, impeding insight into the association of cognitive state and brain activity. To approach these challenges, we introduce the DeepLight framework, which utilizes long short-term memory (LSTM) based DL models to analyze *whole-brain* functional Magnetic Resonance Imaging (fMRI) data. To decode a cognitive state (e.g., seeing the image of a house), DeepLight separates an fMRI volume into a sequence of axial brain slices, which is then sequentially processed by an LSTM. To maintain interpretability, DeepLight adapts the layer-wise relevance propagation (LRP) technique. Thereby, decomposing its decoding decision into the contributions of the single input voxels to this decision. Importantly, the decomposition is performed on the level of single fMRI volumes, enabling DeepLight to study the associations between cognitive state and brain activity on several levels of data granularity, from the level of the group down to the level of single time points. To demonstrate the versatility of DeepLight, we apply it to a large fMRI dataset of the Human Connectome Project. We show that DeepLight outperforms conventional approaches of uni- and multivariate fMRI analysis in decoding the cognitive states and in identifying the physiologically appropriate brain regions associated with these states. We further demonstrate DeepLight's ability to study the fine-grained temporo-spatial variability of brain activity over sequences of single fMRI samples.

## Introduction

Neuroimaging research has recently started collecting large corpora of experimental functional Magnetic Resonance Imaging (fMRI) data, often comprising many hundred individuals (e.g., Poldrack et al., 2013; Van Essen et al., 2013). By collecting these datasets, researchers want to gain insights into the associations between the cognitive states of an individual (e.g., while viewing images or performing a specific task) and the underlying brain activity, while also studying the variability of these associations across the population.

At first sight, the analysis of neuroimaging data thereby seems ideally suited for the application of deep learning (DL; LeCun et al., 2015; Goodfellow et al., 2016) methods, due to the availability of large and structured datasets. Generally, DL can be described as a class of representation-learning methods, with multiple levels of abstraction. At each level, the representation of the input data is transformed by a simple, but non-linear function. The resulting hierarchical structure of non-linear transforms enables DL methods to learn complex functions. It also enables them to identify intricate signals in noisy data, by projecting the input data into a higher-level representation, in which those aspects of the input data that are irrelevant to identify an analysis target are suppressed and those that are relevant are amplified. With this higher-level perspective, DL methods can associate a target variable with variable patterns in the input data. Importantly, DL methods can autonomously learn these projections from the data and therefore do not require a thorough prior understanding of the mapping between input data and analysis target (for a detailed discussion, see LeCun et al., 2015). For these reasons, DL methods seem ideally suited for the analysis of neuroimaging data, where intricate, highly variable patterns of brain activity are hidden in large, high-dimensional datasets and the mapping between cognitive state and brain activity is often unknown.

While researchers have started exploring the application of DL models to neuroimaging data (e.g., Plis et al., 2014; Suk et al., 2014; Nie et al., 2016; Sarraf and Tofighi, 2016; Mensch et al., 2018; Petrov et al., 2018; Yousefnezhad and Zhang, 2018), two major challenges have so far prevented broad DL usage: (1) Neuroimaging data are high dimensional, while containing comparably few samples. For example, a typical fMRI dataset comprises up to a few hundred samples per subject and recently up to several hundred subjects (e.g., Van Essen et al., 2013), while each sample contains several hundred thousand dimensions (i.e., voxels). In such analysis settings, DL models (as well as more traditional machine learning approaches) are likely to suffer from overfitting (by too closely capturing those dynamics that are specific to the training data, so that their predictive performance does not generalize well to new data). (2) DL models have often been considered as non-linear *black box models*, disguising the relationship between input data and decoding decision. Thereby, impeding insight into (and interpretation of) the association between cognitive state and brain activity.

To approach these challenges, we propose the DeepLight framework, which defines a method to utilize long short-term memory (LSTM) based DL architectures (Hochreiter and Schmidhuber, 1997; Donahue et al., 2015) to analyze whole-brain neuroimaging data. In DeepLight, each whole-brain volume is sliced into a sequence of axial images. To decode an underlying cognitive state, the resulting sequence of images is processed by a combination of convolutional and recurrent DL elements. Thereby, DeepLight successfully copes with the high dimensionality of neuroimaging data, while modeling the full spatial dependency structure of whole-brain activity (within and across axial brain slices). Conceptually, DeepLight builds upon the searchlight approach. Instead of moving a small searchlight beam around in space, DeepLight explores brain activity more in-depth, by looking through the full sequence of axial brain slices, before making a decoding decision. To subsequently relate brain activity and cognitive state, DeepLight applies the layer-wise relevance propagation (LRP; Bach et al., 2015; Lapuschkin et al., 2016) method to its decoding decisions. Thereby, decomposing these decisions into the contributions of the single input voxels to each decision. Importantly, the LRP analysis is performed on the level of a single input samples, enabling an analysis on several levels of data granularity, from the level of the group down to the level of single subjects, trials and time points. These characteristics make DeepLight ideally suited to study the fine-grained temporo-spatial distribution of brain activity underlying sequences of single fMRI samples.

Here, we will demonstrate the versatility of DeepLight, by applying it to an openly available fMRI dataset of the Human Connectome Project (Van Essen et al., 2013). In particular, to the data of an N-back task, in which 100 subjects viewed images of either body parts, faces, places or tools in two separate fMRI experiment runs (for an overview, see section Experiment Paradigm and Figure S1). Subsequently, we will evaluate the performance of DeepLight in decoding the four underlying cognitive states (resulting from viewing an image of either of the four stimulus classes) from the fMRI data and identifying the brain regions associated with these states. To this end, we will compare the performance of DeepLight to three representative conventional approaches to the uni- and multivariate analysis of neuroimaging data, with widespread application in the literature. In particular, we will compare DeepLight to the General Linear Model (GLM; Friston et al., 1994), searchlight analysis (Kriegeskorte et al., 2006) and whole-brain Least Absolute Shrinkage Logistic Regression (whole-brain Lasso; Grosenick et al., 2013; Wager et al., 2013). Note that the four analysis approaches differ in the number of voxels they include in their analyses. While the GLM analyses the data of single voxels independent of one another (univariate), the searchlight analysis utilizes the data of clusters of multiple voxels (multivariate) and the whole-brain lasso utilizes the data of all voxels in the brain (whole-brain). In this comparison, we find that DeepLight (1) decodes the cognitive states underlying the fMRI data more accurately than these other approaches, (2) improves its decoding performance better with growing datasets, (3) accurately identifies the physiologically appropriate associations between cognitive states and brain activity, and (4) identifies these associations on multiple levels of data granularity (namely, the level of the group, subject, trial and time point). We also demonstrate DeepLight's ability to study the temporo-spatial distribution of brain activity over a sequence of single fMRI samples.

## Methods

### Experiment Paradigm

Hundred participants performed a version of the N-back task in two separate fMRI runs (for an overview, see Figure S1 and Barch et al., 2013). Each of the two runs (260 s each) consisted of eight task blocks (25 s each) and four fixation blocks (15 s each). Within each run, the four different stimulus types (body, face, place and tool) were presented in separate blocks. Half of the task blocks used a 2-back working memory task (participants were asked to respond “target” when the current stimulus was the same as the stimulus 2 back) and the other half a 0-back working memory task (a target cue was presented at the beginning of each block and the participants were asked to respond “target” whenever the target cue was presented in the block). Each task block consisted of 10 trials (2.5 s each). In each trial, a stimulus was presented for 2 s followed by a 500 ms interstimulus interval (ISI). We were not interested in identifying any effect of the N-back task condition on the evoked brain activity and therefore pooled the data of both N-back conditions.

### FMRI Data Acquisition and Preprocessing

Functional MRI data of 100 unrelated participants for this experiment were provided in a preprocessed format by the Human Connectome Project (HCP S1200 release), WU Minn Consortium (Principal Investigators: David Van Essen and Kamil Ugurbil; 1U54MH091657) funded by the 16 NIH Institutes and Centers that support the NIH Blueprint for Neuroscience Research; and by the McDonnell Center for Systems Neuroscience at Washington University. Whole-brain EPI acquisitions were acquired with a 32 channel head coil on a modified 3T Siemens Skyra with TR = 720 ms, TE = 33.1 ms, flip angle = 52 deg, BW = 2,290 Hz/Px, in-plane FOV = 208 × 180*mm*, 72 slices, 2.0 mm isotropic voxels with a multi-band acceleration factor of 8. Two runs were acquired, one with a right-to-left and the other with a left-to-right phase encoding (for further methodological details on fMRI data acquisition, see Uğurbil et al., 2013).

The Human Connectome Project preprocessing pipeline for functional MRI data (“fMRIVolume”; Glasser et al., 2013) includes the following steps: gradient unwarping, motion correction, fieldmap-based EPI distortion correction, brain-boundary based registration of EPI to structural T1-weighted scan, non-linear registration into MNI152 space, and grand-mean intensity normalization (for further details, see Glasser et al., 2013; Uğurbil et al., 2013). In addition to the minimal preprocessing of the fMRI data that was performed by the Human Connectome Project, we applied the following preprocessing steps to the data for all decoding analyses: volume-based smoothing of the fMRI sequences with a 3 mm Gaussian kernel, linear detrending and standardization of the single voxel signal time-series (resulting in a zero-centered voxel time-series with unit variance) and temporal filtering of the single voxel time-series with a butterworth highpass filter and a cutoff of 128 s, as implemented in Nilearn 0.4.1 (Abraham et al., 2014). In line with previous work (Jang et al., 2017), we further applied an outer brain mask to each fMRI volume. We first identified those voxels whose activity was larger than 5% of the maximum voxel signal within the fMRI volume and then only kept those voxels for further analysis that were positioned between the first and last voxel to fulfill this property in the three spatial dimensions of any functional brain volume of our dataset. This resulted in a brain mask spanning 74 × 92 × 81 voxels (*X* × *Y* × *Z*).

All of our preprocessing was performed by the use of Nilearn 0.4.1 (Abraham et al., 2014). Importantly, we did not exclude any TR of an experiment block of the four stimulus classes from the decoding analyses. However, we removed all fixation blocks from the decoding analyses. Lastly, we split the fMRI data of the 100 subjects contained in the dataset into two distinct training and test datasets (each containing the data of 70 and 30 randomly assigned subjects). All analyses presented throughout the following solely include the data of the 30 subjects contained in the held-out test dataset (if not stated otherwise).

### Data Availability

The data that support the findings of this study are openly available at the ConnectomeDB S1200 Project page of the Human Connectome Project (https://db.humanconnectome.org/data/projects/HCP1200).

### Baseline Methods

#### General Linear Model

The General Linear Model (GLM; Friston et al., 1994) represents a univariate brain encoding model (Naselaris et al., 2011; Kriegeskorte and Douglas, 2018). Its goal is to identify an association between cognitive state and brain activity, by predicting the time series signal of a voxel from a set of experiment predictor:

(1) $$\begin{array}{r} Y=X\beta +\epsilon \end{array}$$

Here, *Y* presents a *T* × *N* dimensional matrix containing the multivariate time series data of *N* voxels and *T* time points. *X* represents the design matrix, which is composed of *T* × *P* data points, where each column represents one of *P* predictors. Typically, each predictor represents a variable that is manipulated during the experiment (e.g., the presentation times of stimuli of one of the four stimulus classes). β represents a *P* × *N* dimensional matrix of regression coefficients. To mimic the blood-oxygen-level dependent (BOLD) response measured by the fMRI, each predictor is first convolved with a hemodynamic response function (HRF; Lindquist et al., 2009), before fitting the β-coefficients to the data. After fitting, the resulting brain map of β-coefficients indicates the estimated contribution of each predictor to the time series signal of each of the *N* voxels. ϵ represents a *T* × *N* dimensional matrix of error terms. Importantly, the GLM analyzes the time series signal of each voxel independently and thereby includes a separate set of regression coefficients for each voxel in the brain.

#### Searchlight Analysis

The searchlight analysis (Kriegeskorte et al., 2006) is a multivariate brain decoding model (Naselaris et al., 2011; Kriegeskorte and Douglas, 2018). Its goal is to identify an association between cognitive state and brain activity, by probing the ability of a statistical classifier to identify the cognitive state from the activity pattern of a small clusters of voxels. To this end, the entire brain is scanned with a sphere of a given radius (the searchlight) and the performance of the classifier in decoding the cognitive states is evaluated at each location, resulting in a brain map of decoding accuracies. These decoding accuracies indicate how much information about the cognitive state is contained in the activity pattern of the underlying cluster of voxels. Here, we used a searchlight radius of 5.6 mm and a linear-kernel Support Vector Machine (SVM) classifier (if not reported otherwise).

Given a training dataset of *T* data points ${\left[y_{t},x_{t}\right]}_{t=1}^{T}$, where *x*_*t*_ represents the activity pattern of a cluster of voxels at time point *t* and *y*_*t*_ the corresponding label, the SVM (Cortes and Vapnik, 1995) is defined as follows:

(2) $$\begin{array}{r} ŷ\left(x\right)=sign\left[\overset{T}{\underset{t=1}{\sum }}\alpha_{t}y_{t}\gamma \left(x,x_{t}\right)+b\right] \end{array}$$

Here, α_*t*_ and *b* are positive constants, whereas γ(*x, x*_*t*_) represents the kernel function. We used a linear kernel function, as implemented in Nilearn 0.4.1 (Abraham et al., 2014). We then defined the decoding accuracy achieved by the searchlight analysis as the maximum decoding accuracy that was achieved at any searchlight location in the brain. Similarly, we used the searchlight location that achieved the highest decoding accuracy to make decoding predictions (for example, to compute the confusion matrix presented in **Figure 2C**).

#### Whole-Brain Least Absolute Shrinkage Logistic Regression

The whole-brain Least Absolute Shrinkage Logistic Regression (or whole-brain lasso; Grosenick et al., 2013; Wager et al., 2013) represents a whole-brain decoding model (Naselaris et al., 2011; Kriegeskorte and Douglas, 2018). It identifies an association between cognitive state and brain activity, by probing the ability of a logistic model to decode the cognitive state from whole-brain activity (with one logistic coefficient β_*i*_ per voxel *i* in the brain). To reduce the risk of overfitting, resulting from the large number of model coefficients, the whole-brain lasso applies Least Absolute Shrinkage regularization to the likelihood function of the logistic model (Tikhonov, 1943; Tibshirani, 1996). Thereby, forcing the logistic model to perform automatic variable selection during parameter estimation, resulting in sparse coefficient estimates (i.e., by forcing many coefficient estimates to be exactly 0). In particular, the optimization problem of the whole-brain lasso can be defined as follows (again, *N* denotes the number of voxels in the brain, *T* the number of fMRI sampling time points and ${\left[y_{t},x_{t}\right]}_{t=1}^{T}$ the set of class labels and voxel values of each fMRI sample):

(3) $$\begin{array}{l} \underset{\beta }{min}\{\text{−}\sum_{t=1}^{T}\left[y_{t}\text{log}\sigma \left(\beta^{T}x_{t}\right)\text{+}\left(\text{1-}y_{t}\right)\log\left(\text{1-}\sigma \left(\beta^{T}x_{t}\right)\right)\right]\\ \;+\text{λ}\sum_{i=1}^{N}\left|\beta_{i}\right|\} \end{array}$$

Here, λ represents the strength of the L1 regularization term (with larger values indicating stronger regularization), whereas σ represents the logistic model:

(4) $$\begin{array}{r} \sigma \left(x\right)=\frac{1}{1+e^{-x}} \end{array}$$

For each voxel *i* in the brain, the resulting set of coefficient estimates β, indicates the contribution of the activity of this voxel to the decoding decision σ(*x*_*t*_) of the logistic model for a whole-brain fMRI sample *x*_*t*_ at time point *t*. Over the recent years, the whole-brain lasso, as well as closely related decoding approaches (e.g., McIntosh and Lobaugh, 2004; Ryali et al., 2010; Gramfort et al., 2013), have found widespread application throughout the neuroscience literature (e.g., Wager et al., 2013; Chang et al., 2015).

### DeepLight Framework

#### Deep Learning Model

**Figure 1:**
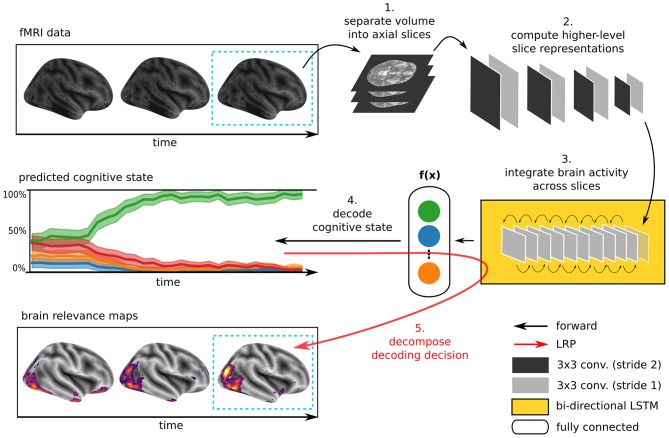
Illustration of the DeepLight approach. A whole-brain fMRI volume is sliced into a sequence of axial images. These images are then passed to a DL model consisting of a convolutional feature extractor, an LSTM and an output unit. First, the convolutional feature extractor reduces the dimensionality of the axial brain slices through a sequence of eight convolution layers. The resulting sequence of higher-level slice representations is then fed to a bi-directional LSTM, modeling the spatial dependencies of brain activity within and across brain slices. Lastly, the DL model outputs a decoding decision about the cognitive state underlying the fMRI volume, through a softmax output layer with one output neuron per cognitive state in the data. Once the prediction is made, DeepLight utilizes the LRP method to decompose the prediction into the contributions (or relevance) of the single input voxels to the prediction. Thereby, enabling an analysis of the association between fMRI data and cognitive state.

The DL model underlying DeepLight consists of three distinct computational modules, namely a feature extractor, an LSTM, and an output unit (for an overview, see Figure 1). First, DeepLight separates each fMRI volume into a sequence of axial brain slices. These slices are then processed by a convolutional feature extractor (LeCun and Bengio, 1995), resulting in a sequence of higher-level, and lower-dimensional, slice representations. These higher-level slice representations are fed to an LSTM (Hochreiter and Schmidhuber, 1997), integrating the spatial dependencies of the observed brain activity within and across axial brain slices. Lastly, the output unit makes a decoding decision, by projecting the output of the LSTM into a lower-dimensional space, spanning the cognitive states in the data. Here, a probability for each cognitive state is estimated, indicating whether the input fMRI volume belongs to each of these states. This combination of convolutional and recurrent DL elements is inspired by previous research, showing that it is generally well-suited to learn the spatial dependency structure of long sequences of input data (Donahue et al., 2015; McLaughlin et al., 2016; Marban et al., 2019). Importantly, the DeepLight approach is not dependent on any specific architecture of each of these three modules. The DL model architecture described in the following is exemplary and derived from previous work (Marban et al., 2019). Further research is needed to explore the effect of specific module architectures on the performance of DeepLight.

The feature extractor used here was composed of a sequence of eight convolution layers (LeCun and Bengio, 1995). A convolution layer consists of a set of kernels (or filters) *w* that each learn local features of the input image *a*. These local features are then convolved over the input, resulting in an activation map *h*, indicating whether a feature is present at each given location of the input:

(5) $$\begin{array}{r} h_{i,j}=g\left(\overset{m}{\underset{k=0}{\sum }}\overset{n}{\underset{l=0}{\sum }}w_{k,l}a_{\left(i-k\right),\left(j-l\right)}\right) \end{array}$$

Here, *b* represents the bias of the kernel, while *g* represents the activation function. *k* and *l* represent the row and column index of the kernel matrix, whereas *i* and *j* represent the row and column index of the activation map.

Generally, lower-level convolution kernels (that are close to the input data) have small receptive fields and are only sensitive to local features of small patches of the input data (e.g., contrasts and orientations). Higher-level convolution kernels, on the other hand, act upon a higher-level representation of the input data, which has already been transformed by a sequence of preceding lower-level convolution kernels. Higher-level kernels thereby integrate the information provided by lower-level convolution kernels, allowing them to identify larger and more complex patterns in the data. We specified the sequence of convolution layers as follows (see Figure 1): conv3-16, conv3-16, conv3-16, conv3-16, conv3-32, conv3-32, conv3-32, conv3-32 [notation: conv(kernel size) - (number of kernels)]. All convolution kernels were activated through a rectified linear unit function:

(6) $$\begin{array}{r} g\left(z\right)=max\left(0,z\right) \end{array}$$

Importantly, all kernels of the even-numbered convolution layers were moved over the input fMRI slice with a stride size of one voxel and all kernels of odd-numbered layers with a stride size of two voxels. The stride size determines the dimensionality of the outputted slice representation. An increasing stride indicates more distance between the application of the convolution kernels to the input data. Thereby, reducing the dimensionality of the output representation at the cost of a decreasing sensitivity to differences in the activity patterns of neighboring voxels. Yet, the activity patterns of neighboring voxels are known to be highly correlated, leading to an overall low risk of information loss through a reasonable increase in stride size. To avoid any further loss of dimensionality between the convolution layers, we applied zero-padding. Thereby, adding zeros to the borders of the inputs to each convolution layer so that the outputs of the convolution layers have the same dimensionality as their inputs, if a stride of one voxel is applied, and only decrease in size, when a larger stride is used. The sequence of eight convolution layers thereby resulted in a 960-dimensional representation of each volume slice.

To integrate the information provided by the resulting sequence of slice representations into a higher-level representation of the observed whole-brain activity, DeepLight applies a bi-directional LSTM (Hochreiter and Schmidhuber, 1997), containing two independent LSTM units. Each of the two LSTM units iterates through the entire sequence of input slices, but in reverse order (one from bottom-to-top and the other from top-to-bottom). An LSTM unit contains a hidden cell state *C*, storing information over an input sequence of length *S* with elements *a*_*s*_ and outputs a vector *h*_*s*_ for each input at sequence step *s*. The unit has the ability to add and remove information from *C* through a series of gates. In a first step, the LSTM unit decides what information from the cell state *C* is removed. This is done by a fully-connected logistic layer, the forget gate *f*:

(7) $$\begin{array}{r} f_{t}=\sigma \left(W_{f}a_{s}+U_{f}h_{s-1}+b_{f}\right) \end{array}$$

Here, σ indicates the logistic function (see Equation 4), [*W, U*] the gate's weight matrices and *b* the gate's bias. The forget gate outputs a number between 0 and 1 for each entry in the cell state *C* at the previous sequence step *s*−1. Next, the LSTM unit decides what information is going to be stored in the cell state. This operation contains two elements: the input gate *i*, which decides which values of *C*_*s*_ will be updated, and a tanh layer, which creates a new vector of candidate values $C_{s}^{\prime }$:

(8) $$\begin{array}{r} i_{s}=\sigma \left(W_{i}a_{s}+U_{i}h_{s-1}+b_{i}\right) \end{array}$$

(9) $$\begin{array}{r} C_{s}^{\prime }=\tanh\left(W_{c}a_{s}+U_{c}h_{s-1}+b_{c}\right) \end{array}$$

(10) $$\begin{array}{r} \tanh\left(z\right)=\frac{e^{z}-e^{-z}}{e^{z}+e^{-z}} \end{array}$$

Subsequently, the old cell state *C*_*s*−1_ is updated into the new cell state

(11) $$\begin{array}{r} C_{s}=f_{s}\cdot C_{s-1}+i_{s}\cdot C_{s}^{\prime } \end{array}$$

Lastly, the LSTM computes its output *h*_*s*_. Here, the output gate *o*, decides what part of *C*_*s*_ will be outputted. Subsequently, *C*_*s*_ is multiplied by another *tanh* layer to make sure that *h*_*s*_ is scaled between −1 and 1:

(12) $$\begin{array}{r} o_{s}=\sigma \left(W_{o}a_{s}+U_{o}h_{s-1}+b_{o}\right) \end{array}$$

(13) $$\begin{array}{r} h_{s}=o_{s}\cdot tanh\left(C_{s}\right) \end{array}$$

Each of the two LSTM units in our DL model contained 40 output neurons. To make a decoding decision, both LSTM units pass their output for the last sequence element to a fully-connected softmax output layer. The output unit contains one neuron per cognitive state in the data and assigns a probability to each of the *K* (here, *K* = 4) states, indicating the probability that the current fMRI sample belongs to this state:

(14) $$\begin{array}{r} \sigma =\frac{e^{z_{j}}}{\sum_{k=1}^{K}e^{z_{k}}},withj=1,\ldots ,K \end{array}$$

#### Layer-Wise Relevance Propagation in the DeepLight Framework

To relate the decoded cognitive state and brain activity, DeepLight utilizes the Layer-Wise Relevance Propagation (LRP; Bach et al., 2015; Montavon et al., 2017; Lapuschkin et al., 2019) method. The goal of LRP is to identify the contribution of a single dimension *d* of an input *a* (with dimensionality *D*) to the prediction *f*(*a*) that is made by a linear or non-linear classifier *f*. We denote the contribution of a single dimension as its relevance *R*_*d*_. One way of decomposing the prediction *f*(*a*) is by the sum of the relevance values of each dimension of the input:

(15) $$\begin{array}{r} f\left(a\right)\approx \overset{D}{\underset{d=1}{\sum }}R_{d} \end{array}$$

Qualitatively, any *R*_*d*_ < 0 can be interpreted as evidence against the presence of a classification target, while *R*_*d*_ > 0 denotes evidence for the presence of the target. Importantly, LRP assumes that *f*(*a*) > 0 indicates evidence for the presence of a target.

Let's assume the relevance $R_{j}^{\left(l\right)}$ of a neuron *j* at network layer *l* for the prediction *f*(*a*) is known. We would like to decompose this relevance into the messages $R_{i\leftarrow j}^{\left(l-1,l\right)}$ that are sent to those neurons *i* in layer *l*−1 which provide the inputs to neuron *j*:

(16) $$\begin{array}{r} R_{j}^{\left(l\right)}=\underset{i\epsilon \left(l\right)}{\sum }R_{i\leftarrow j}^{\left(l-1,l\right)} \end{array}$$

While the relevance of the output neuron at the last layer *L* is defined as $R_{d}^{\left(L\right)}=f\left(a\right)$, the dimension-wise relevance scores on the input neurons are given by $R_{d}^{\left(1\right)}$. For all weighted connections of the DL model in between (see Equations 5, 7, 8, 9, and 12), DeepLight defines the messages $R_{i\leftarrow j}^{\left(l-1,l\right)}$ as follows:

(17) $$\begin{array}{r} R_{i\leftarrow j}^{\left(l-1,l\right)}=\frac{z_{ij}}{z_{j}+\epsilon \cdot sign\left(z_{j}\right)}R_{j}^{\left(l\right)} \end{array}$$

Here, $z_{ij}=a_{i}^{\left(l-1\right)}w_{ij}^{\left(l-1,l\right)}$ (*w* indicating the coefficient weight and *a* the input) and $z_{j}=\underset{i}{\sum }z_{ij}$, while ϵ represents a stabilizer term that is necessary to avoid numerical degenerations when *z*_*j*_ is close to 0 (we set ϵ = 0.001).

Importantly, the LSTM also applies another type of connection, which we refer to as multiplicative connection (see Equations 11 and 13). Let *z*_*j*_ be an upper-layer neuron whose value in the forward pass is computed by multiplying two lower-layer neuron values *z*_*g*_ and *z*_*s*_ such that *z*_*j*_ = *z*_*g*_·*z*_*s*_. These multiplicative connections occur when we multiply the outputs of a *gate* neuron, whose values range between 0 and 1, with an instance of the hidden cell state, which we will call *source* neuron. For these types of connections, we set the relevances of the gate neuron $R_{g}^{\left(l-1\right)}=0$ and the relevances of the source neuron $R_{s}^{\left(l-1\right)}=R_{j}^{\left(l\right)}$, where $R_{j}^{\left(l\right)}$ denotes the relevances of the upper layer neuron *z*_*j*_ (as proposed in Arras et al., 2017). The reasoning behind this rule is that the gate neuron already decides in the forward pass how much of the information contained in the source neuron should be retained to make the classification. Even if this seems to ignore the values of the neurons *z*_*g*_ and *z*_*s*_ for the redistribution of relevance, these are actually taken into account when computing the value $R_{j}^{\left(l\right)}$ from the relevances of the next upper-layer neurons to which *z*_*j*_ is connected by the weighted connections. We refer the reader to Samek et al. (2018) and Montavon et al. (2018) for more information about explanation methods.

In the context of this work, we decomposed the predictions of DeepLight for the actual cognitive state underlying each fMRI sample, as we were solely interested in understanding what DeepLight used as evidence in favor of the presence of this state. We also restricted the LRP analysis to those brain samples that the DL model classified correctly, because we can only assume that the DL model has learned a meaningful mapping between brain data and cognitive state, if it is able to accurately decode the cognitive state.

#### DeepLight Training

We iteratively trained DeepLight through backpropagation (Rumelhart et al., 1986) over 60 epochs by the use of the ADAM optimization algorithm as implemented in tensorflow 1.4 (Abadi et al., 2016). To prevent overfitting, we applied dropout regularization to all network layers (Srivastava et al., 2014), global gradient norm clipping (with a clipping threshold of 5; Pascanu et al., 2013), as well as an early stopping of the training (for an overview of training statistics, see Figure S2). During the training, we set the dropout probability to 50% for all network layers, except for the first four convolution layers, where we reduced the dropout probability to 30% for the first two layers and 40% for the third and fourth layer. Each training epoch was defined as a complete iteration over all samples in the training dataset (see section FMRI Data Acquisition and Preprocessing). We used a learning rate of 0.0001 and a batch size of 32. All network weights were initialized by the use of a normal-distributed random initialization scheme (Glorot and Bengio, 2010). The DL model was written in tensorflow 1.4 (Abadi et al., 2016) and the interprettensor library (https://github.com/VigneshSrinivasan10/interprettensor).

#### DeepLight Brain Maps

To generate a set of subject-level brain maps with DeepLight, we first decomposed the decoding decisions of DeepLight for each correctly classified fMRI sample of a subject with the LRP method (see section Layer-Wise Relevance Propagation in the DeepLight Framework). Importantly, we restricted the LRP analysis to those fMRI samples that were collected 5–15 s after the onset of the experiment block, as we expect the HRF (Lindquist et al., 2009) to be strongest within this time period. To then aggregate the resulting set of relevance maps for each decomposed fMRI sample within each cognitive state, we smoothed each relevance map with a 3 mm FWHM Gaussian kernel and averaged all relevance volumes belonging to a cognitive state, resulting in one brain map per subject and cognitive state. Group-level brain maps were then obtained, by averaging these subject-level brain maps for all subjects in the held-out test dataset within each cognitive state, resulting in one group-level brain map per cognitive state.

## Results

### DeepLight Accurately Decodes Cognitive States From fMRI Data

A key prerequisite for the DeepLight analysis (as well as all other decoding analyses) is that it achieves reasonable performance in the decoding task at hand. Only then we can assume that it has learned a meaningful mapping from the fMRI data to the cognitive states and interpret the resulting brain maps as informative about these states.

**Figure 2:**
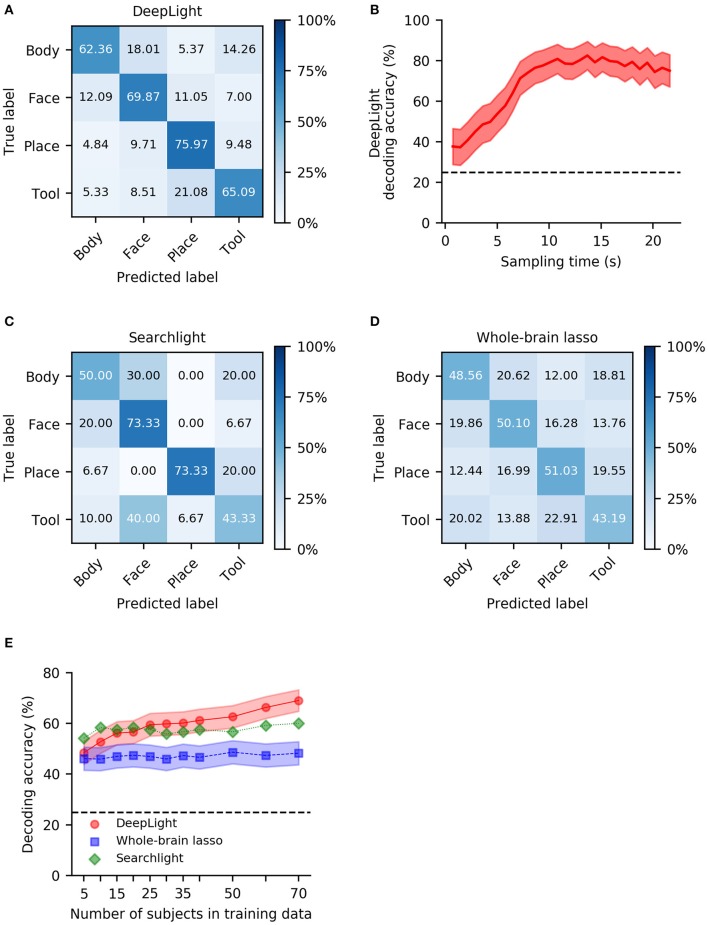
Group-level decoding performance of DeepLight, the searchlight analysis and whole-brain lasso. **(A)** Confusion matrix of DeepLight's decoding decisions. **(B)** Average decoding performance of DeepLight over the course of an experiment block. **(C,D)** Confusion matrix for the decoding decisions of the group-level searchlight analysis **(C)** and whole-brain lasso **(D)**. **(E)** Average decoding accuracy of the searchlight (green), whole-brain lasso (blue) and DeepLight (red), when these are repeatedly trained on a subset of the subjects from the full training dataset. Black dashed horizontal lines indicate chance level.

Overall, DeepLight accurately decoded the cognitive states underlying 68.3% of the fMRI samples in the held-out test dataset (62.36, 69.87, 75.97, 65.09 for body, face, place and tool, respectively; Figure 2A). It generally performed best at discriminating the body and place (5.1% confusion in the held-out data), face and tool (7.8% confusion in the held-out data), body and tool (9.8% confusion in the held-out data) and face and place (10.4% confusion in the held-out data) stimuli from the fMRI data, while it did not perform as well in discriminating place and tool and body and face stimuli (15% confusion in the held-out data, respectively).

Note that DeepLight's performance in decoding the four cognitive states from the fMRI data varied over the course of an experiment block (Figure 2B). DeepLight performed best in the middle and later stages of the experiment block, where the average decoding accuracy reaches 80%. This finding is generally in line with the temporal evolution of the hemodynamic response function (HRF; Lindquist et al., 2009) measured by the fMRI (the HRF is known to be strongest 5–10 s after to the onset of the underlying neuronal activity).

To further evaluate DeepLight's performance in decoding the cognitive states from the fMRI data, we compared its performance in decoding these states to the searchlight analysis and whole-brain lasso. For simplicity, we sub-divided this comparison into a separate analysis on the group- and subject-level.

#### Group-Level

For the group-level comparison, we trained the searchlight analysis and whole-brain lasso on the data of all 70 subjects contained in the training dataset (for details on the fitting procedures, see section Parameter Estimation of the Baseline Methods in Supplementary Information). Subsequently, we evaluated their performance in decoding the cognitive states in the full held-out test data.

DeepLight clearly outperformed the other approaches in decoding the cognitive states. While the searchlight analysis achieved an average decoding accuracy of 60% (Figure 2C) and the whole-brain lasso an average decoding accuracy of 47.97% (Figure 2D), DeepLight improved upon these performances by 8.3% [*t*_(29)_ = 5.80, *p* < 0.0001] and 20.33% [*t*_(29)_ = 13.39, *p* < 0.0001], respectively.

All three decoding approaches generally performed best at discriminating face and place stimuli from the fMRI data (Figures 2A,C,D). Similar to DeepLight, the searchlight analysis and whole-brain lasso also performed well at discriminating body and place stimuli (3.3 and 12.2% confusion for the searchlight analysis and whole-brain lasso, respectively, Figures 2C,D), while they also had more difficulties discriminating body and face stimuli from the fMRI data (25 and 20.2% confusion for the searchlight analysis and whole-brain lasso, respectively, Figures 2C,D).

A key premise of DL methods, when compared to more traditional decoding approaches, is that their decoding performance improves better with growing datasets. To test this, we repeatedly trained all three decoding approaches on a subset of the training dataset (including the data of 5, 10, 15, 20, 25, 30, 35, 40, 50, 60, and 70 subjects), and validated their performance at each iteration on the full held-out test data (Figure 2E). Overall, the decoding performance of DeepLight increased by 0.27% [*t*_(10)_ = 10.9, *p* < 0.0001] per additional subject in the training dataset, whereas the performance of the whole-brain lasso increased by 0.03% [*t*_(10)_ = 3.02, *p* = 0.015] and the performance of the searchlight analysis only marginally increased by 0.04% [*t*_(10)_ = 2.08, *p* = 0.067]. Nevertheless, the searchlight analysis outperformed DeepLight in decoding the cognitive states from the data when only little training data were available (here, 10 or less subjects [*t*_(29)_ = −4.39, *p* < 0.0001]. The decoding advantage of DeepLight, on the other hand, came to light when the data of 50 or more subjects were available in the training dataset [*t*_(29)_ = 3.82, *p* = 0.0006]. DeepLight consistently outperformed the whole-brain lasso, when it was trained on the data of at least 10 subjects [*t*_(29)_ = 5.32, *p* = 0.0045].

#### Subject-Level

For the subject-level comparison, we first trained both, the searchlight analysis and whole-brain lasso on the fMRI data of the first experiment run of a subject from the held-out test dataset (for an overview of the training procedures, see section Parameter Estimation of the Baseline Methods in Supplementary Information). We then used the data of the second experiment run of the same subject to evaluate their decoding performance (by predicting the cognitive states underlying each fMRI sample of the second experiment run). Importantly, we also decoded the same fMRI samples with DeepLight. Note that DeepLight, in comparison to the other approaches, did not see any data of the subject during the training, as it was solely trained on the data of the 70 subjects in the training dataset (see section DeepLight Training).

**Figure 3:**
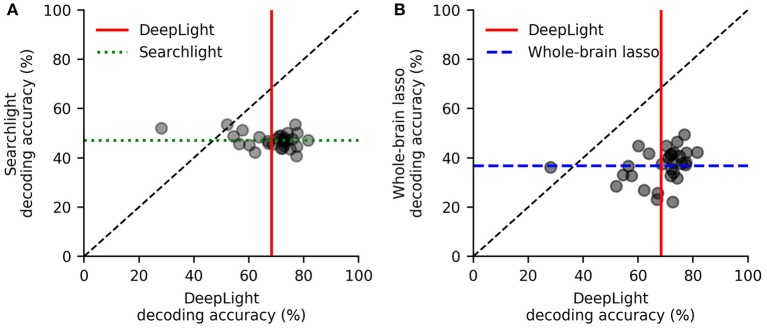
Subject-level decoding performance comparison of DeepLight (red) to the searchlight analysis (**A**; green) and whole-brain lasso (**B**; blue). Black scatter points indicate the average decoding accuracy for a subject. Colored lines indicate the average decoding accuracy across all 30 test subjects.

DeepLight clearly outperformed the other decoding approaches, by decoding the cognitive states more accurately for 28 out of 30 subjects, when compared to the searchlight analysis (while the searchlight analysis achieved an average decoding accuracy of 47.2% across subjects, DeepLight improved upon this performance by 22.4%, with an average decoding accuracy of 69.3%, *t*_(29)_ = 11.28, *p* < 0.0001; Figure 3A), and for 29 out of 30 subjects, when compared to the whole-brain lasso (while the whole-brain lasso achieved an average decoding accuracy of 37% across subjects, DeepLight improved upon this performance by 32%; *t*_(29)_ = 15.74, *p* < 0.0001; Figure 3B).

To further ascertain that the observed differences in decoding performance between the searchlight and DeepLight did not result from the linearity contained in the Support Vector Machine (SVM; Cortes and Vapnik, 1995) of the searchlight analysis, we replicated our subject-level searchlight analysis, by the use of a non-linear radial basis function kernel (RBF; Cortes and Vapnik, 1995; Müller et al., 2001; Schölkopf and Smola, 2002) SVM (Figure S3). However, the decoding accuracies achieved by the RBF-kernel SVM were not meaningfully different from those of the linear-kernel SVM [*t*_(29)_ = −1.75, *p* = 0.09].

Lastly, we also compared the subject-level decoding performance of the whole-brain lasso to that of a recently proposed extension of this approach (TV-L1, for methodological details see Gramfort et al., 2013). The TV-L1 approach combines the Least Absolute Shrinkage Regularization (L1; see Equation 3) of the whole-brain lasso with an additional Total-Variation (TV) penalty (Michel et al., 2011), to better account for the spatial dependency structure of fMRI data. Yet, we found that the whole-brain lasso performed better at decoding the cognitive states from the subject-level fMRI data than TV-L1 [*t*_(29)_ = 3.79, *p* = 0.0007; see Figure S4].

### DeepLight Identifies Physiologically Appropriate Associations Between Cognitive States and Brain Activity

**Figure 4:**
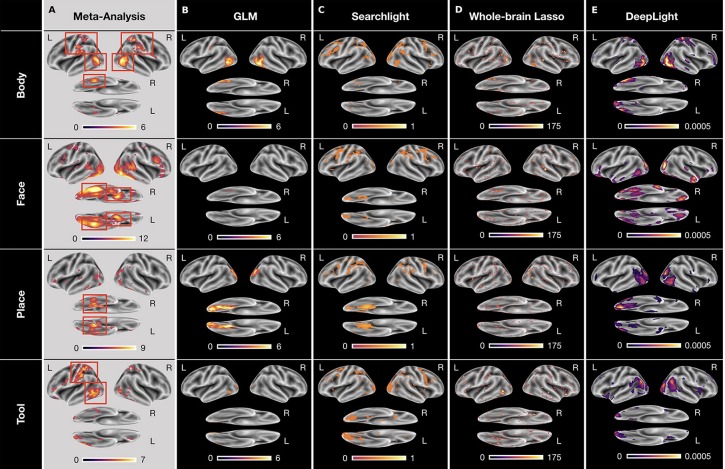
Group-level brain maps for each cognitive state and analysis approach: **(A)** Results of a NeuroSynth meta-analysis for the terms “body,” “face,” “place,” and “tools.” The brain maps were thresholded at an expected false discovery rate of 0.01. Red boxes highlight the regions-of-interest for each cognitive state. **(B)** Results of the GLM group-level analysis. The brain maps of the GLM analysis were thresholded at an expected false discovery rate of 0.1. **(C–E)** Results of the group-level searchlight analysis **(C)**, whole-brain lasso **(D)**, and DeepLight **(E)**. The brain maps of the searchlight analysis, whole-brain lasso, and DeepLight were thresholded at the 90th percentile of their values. Note that the values of the brain maps are on different scales between analysis approaches, due to their different statistical nature. All brain maps are projected onto the inflated cortical surface of the FsAverage5 surface template (Fischl, 2012).

**Figure 5:**
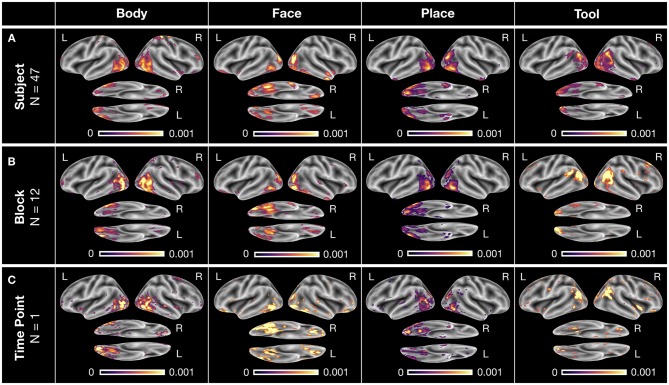
Exemplary DeepLight brain maps for each of the four cognitive states on different levels of data granularity for a single subject. All brain maps belong to the subject with the highest decoding accuracy in the held-out test dataset. **(A)** Average relevance maps for all correctly classified TRs of the subject (with an average of 47 TRs per cognitive state). **(B)** Average relevance maps for all correctly classified TRs of the first experiment block of each cognitive state in the first experiment run (with an average of 12 TRs per cognitive state). **(C)** Exemplar relevance maps for a single TR of the first experiment block of each cognitive state in the first experiment run. All relevance maps were thresholded at the 90th percentile of their values and projected onto the inflated cortical surface of the FsAverage5 surface template (Fischl, 2012).

**Figure 6:**
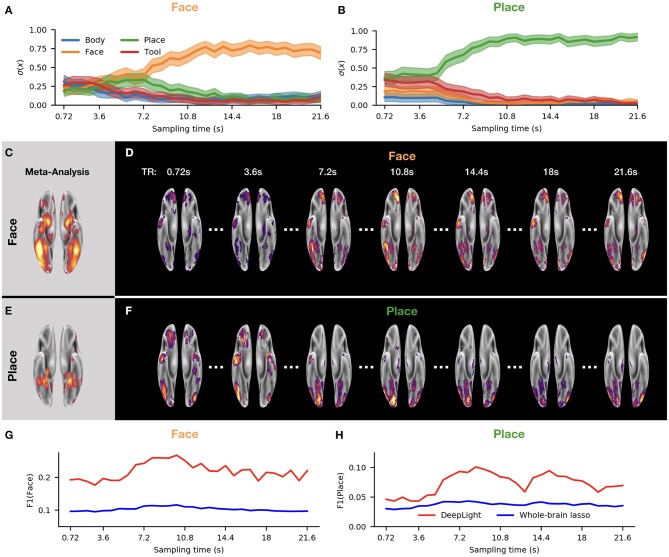
DeepLight analysis of the temporo-spatial distribution of brain activity in the first experiment block of the face and place stimulus classes in the second experiment run of the held-out test dataset. **(A,B)** Average predicted probability that the fMRI data collected at each sampling time point belongs to each of the four cognitive states. **(C,E)** Results of a meta-analysis with the NeuroSynth database for the face and place stimulus classes (for details on the meta-analysis, see section NeuroSynth in Supplementary Information). **(D,F)** Group-level brain maps for seven fMRI sampling time points from the experiment block. Each group-level brain map at each time point is computed as an average over the relevance maps of each subject for this time point. Each group-level brain map is thresholded at the 90th percentile of its values. All brain maps are projected onto the inflated cortical surface of the FsAverage5 surface template (Fischl, 2012). **(G,H)** F1-score for each group-level brain map at each sampling time point of the experiment block. The F1-score quantifies the similarity between the group-level brain map and the results of the meta-analysis **(C,E)** (for further details on the F1-score, see section Subject-Level and section NeuroSynth in Supplementary Information). Red indicates the results of the F1-score comparison for the brain maps of DeepLight, whereas blue indicates the results of this comparison for the brain maps of the whole-brain lasso analysis (for further details on the F1-comparison for the whole-brain lasso analysis, see section DeepLight's Relevance Patterns Resemble Temporo-Spatial Variability of Brain Activity Over Sequences of Single fMRI Samples).

Our previous analyses have shown that DeepLight has learned a meaningful mapping between the fMRI data and cognitive states, by accurately decoding these states from the data. Next, we therefore tested DeepLight's ability to identify the brain areas associated with the cognitive states, by decomposing its decoding decisions with the LRP method (see section DeepLight Framework). Subsequently, we compared the resulting brain maps of DeepLight to those of the GLM, searchlight analysis and whole-brain lasso. Again, we sub-divided this comparison into a separate analysis on the group- and subject-level. Note that due to the diverse statistical nature of the three baseline approaches, the values of their brain maps are on different scales and have different statistical interpretations (for methodological details, see section Baseline Methods). Further, all depicted brain maps in Figures 4–**6** are projected onto the inflated cortical surface of the FsAverage5 surface template (Fischl, 2012) for better visibility.

To evaluate the quality of the brain maps resulting from each analysis approach, we performed a meta-analysis of the four cognitive states with NeuroSynth (for details on NeuroSynth, see section NeuroSynth in Supplementary Information and Yarkoni et al., 2011). NeuroSynth provides a database of mappings between cognitive states and brain activity, based on the empirical neuroscience literature. Particularly, the resulting brain maps used here indicate whether the probability that an article reports a specific brain activation is different, when it includes a specific term (e.g., “face”) compared to when it does not. With this meta-analysis, we defined a set of *regions-of-interest* (ROIs) for each cognitive state (as defined by the terms “body,” “face,” “place,” and “tools”), in which we would expect the various analysis approaches to identify a positive association between the cognitive state and brain activity (for an overview, see Figure 4A). These ROIs were defined as follows: the upper parts of the middle and inferior temporal gyrus, the postcentral gyrus, as well as the right fusiform gyrus for the body state, the fusiform gyrus (also known as the fusiform face area FFA; Haxby et al., 2001; Heekeren et al., 2004) and amygdala for the face state, the parahippocampal gyrus (or parahippocampal place area PPA; Haxby et al., 2001; Heekeren et al., 2004) for the place state and the upper left middle and inferior temporal gyrus as well as the left postcentral gyrus for the tool state.

To ensure comparability with the results of the meta-analysis, we restricted all analyses of brain maps to the estimated positive associations between brain activity and cognitive states (i.e., positive relevance values as well as positive GLM and whole-brain lasso coefficients, see section Baseline Methods and section Parameter Estimation of the Baseline Methods in Supplementary Information). A negative Z-value in the meta-analysis indicates a lower probability that an article reports a specific brain activation when it includes a specific term, compared to when it does not include the term. A negative value in the meta-analysis is therefore conceptually different to negative values in the brain maps of our analyses (e.g., negative relevance values or negative whole-brain lasso coefficients). These can generally be interpreted as evidence against the presence of a cognitive state, given the specific set of cognitive states in our dataset (e.g., a negative relevance indicates evidence for the presence of any of the other cognitive states considered).

#### Group-Level

To determine the voxels that each analysis approach associated with a cognitive state, we defined a threshold for the values of each group-level brain map, indicating those voxels that are associated most strongly with the cognitive state. For the GLM analysis, we thresholded all *P*-values at an expected false discovery rate (Benjamini and Hochberg, 1995; Genovese et al., 2002) of 0.1 (Figure 4B). Similarly, for all decoding analyses, we thresholded each brain map at the 90th percentile of its values (Figures 4C–E). For the whole-brain lasso and DeepLight, the remaining 10 percent of values indicate those brain regions whose activity these approaches generally weight most in their decoding decisions. For the searchlight analysis, the remaining 10 percent of values indicate those brain regions in which the searchlight analysis achieved the highest decoding accuracy.

All analysis approaches correctly associated activity in the upper parts of the middle and inferior temporal gyrus with body stimuli. The GLM, whole-brain lasso and DeepLight also correctly associated activity in the right fusiform gyrus with body stimuli. Only DeepLight correctly associated activity in the postcentral gyrus with these stimuli. The GLM, whole-brain lasso and DeepLight further all correctly associated activity in the right FFA with face stimuli. None of the approaches, however, associated activity in the left FFA with face stimuli. Interestingly, the searchlight analysis did not associate the FFA with face stimuli at all. All analysis approaches also correctly associated activity in the PPA with place stimuli. Lastly, for tool stimuli, the GLM and whole-brain lasso correctly associated activity in the left inferior temporal sulcus with stimuli of this class. The searchlight analysis and whole-brain lasso only did so marginally. None of the approaches associated activity in the left postcentral gyrus with tool stimuli.

Overall, DeepLight's group-level brain maps accurately associated each of the ROIs with their respective cognitive states. Interestingly, DeepLight also associated a set of additional brain regions with the face and tool stimulus classes that were not identified by the other analysis approaches (see Figure 4E). For face stimuli, these regions are the orbitofrontal cortex and temporal pole. While the temporal pole has been shown to be involved in the ability of an individual to infer the desires, intentions and beliefs of others (*theory-of-mind*; for a detailed review, see Olson et al., 2007), the orbitofrontal cortex has been associated with the processing of emotions in the faces of others (for a detailed review, see Adolphs, 2002). For tool stimuli, DeepLight additionally utilized the activity of the temporoparietal junction (TPJ) to decode these stimuli. The TPJ has been shown to be associated with the ability of an individual to discriminate self-produced actions and the actions produced by others and is generally regarded of as a central hub for the integration of body-related information (for a detailed review, see Decety and Grèzes, 2006). Although it is not clear why only DeepLight associated these brain regions with the face and tool stimulus classes, their assumed functional roles do not contradict this association.

#### Subject-Level

The goal of the subject-level analysis was to test the ability of each analysis approach to identify the physiologically appropriate associations between brain activity and cognitive state on the level of each individual.

To quantify the similarity between the subject-level brain maps and the results of the meta-analysis, we defined a similarity measure. Given a target brain map (e.g., the results of our meta-analysis), this measure tests for each voxel in the brain whether a source brain map (e.g., the results of our subject-level analyses) correctly associates this voxel's activity with the cognitive state (true positive), falsely associates the voxel's activity with the cognitive state (false positives) or falsely does not associate the voxel's activity with the cognitive state (false negatives). Particularly, we derived this measure from the well-known F1-score in machine learning (see section F1-Score in Supplementary Information as well as Goutte and Gaussier, 2005). The benefit of the F1-score, when compared to simply computing the ratio of correctly classified voxels in the brain, is that it specifically considers the brain map's precision and recall and is thereby robust to the overall size of the ROIs in the target brain map. Here, precision describes the fraction of true positives from the total number of voxels that are associated with a cognitive state in the source brain map. Recall, on the other hand, describes the fraction of true positives from the overall number of voxels that are associated with a cognitive state in the target brain map. Generally, an F1-score of 1 indicates that the brain map has both, perfect precision and recall with respect to the target, whereas the F1-score is worst at 0.

To obtain an F1-score for each subject-level brain map (for details on the estimation of subject-level brain maps with the three baseline analysis approaches, see section Parameter Estimation of the Baseline Methods in Supplementary Information), we again thresholded each individual brain map. For the GLM, we defined all voxels with *P* > 0.005 (uncorrected) as not associated with the cognitive state and all others as associated with the cognitive state. For the searchlight analysis, whole-brain lasso and DeepLight, we defined all voxels with a value below the 90th percentile of the values within the brain map as not associated with the cognitive state and all others as associated with the cognitive state.

Overall, DeepLight's subject-level brain maps had meaningfully larger F1-scores for the body, face and place stimulus classes, when compared to those of the GLM [*t*_(29)_ = 10.46, *p* < 0.0001 for body stimuli, Figure S5A; *t*_(29)_ = 13.04, *p* < 0.0001 for face stimuli, Figure S5D; *t*_(29)_ = 9.26, *p* < 0.0001 for place stimuli, Figure S5G], searchlight analysis [*t*_(29)_ = 13.26, *p* < 0.0001 for body stimuli, Figure S5B; *t*_(29)_ = 8.57, *p* < 0.0001 for face stimuli, Figure S5E; *t*_(29)_ = 4.25, *p* = 0.0002, for place stimuli, Figure S5H], and whole-brain lasso [*t*_(29)_ = 20.93, *p* < 0.0001 for body stimuli, Figure S5C; *t*_(29)_ = 48.32, *p* < 0.0001 for face stimuli, Figure S5F; *t*_(29)_ = 22.43, *p* < 0.0001, for place stimuli, Figure S5I]. For tool stimuli, the GLM and searchlight generally achieved higher subject-level F1-scores than DeepLight [*t*_(29)_ = −8.19, *p* < 0.0001, Figure S5J; *t*_(29)_ = −4.39, *p* = 0.0001, Figure S5K for the GLM and searchlight, respectively], whereas DeepLight outperformed the whole-brain lasso analysis [*t*_(29)_ = 18.31, *p* < 0.0001, Figure S5L].

To ascertain that the results of this comparison were not dependent on the thresholds that we chose, we replicated the comparison for each combination of the 85th, 90th, and 95th percentile threshold for the brain maps of the searchlight analysis, whole-brain lasso and DeepLight, as well as a P-threshold of 0.05, 0.005, 0.0005, and 0.00005 for the brain maps of the GLM. Within all combinations of percentile values and P-thresholds, the presented results of the F1-comparison were generally stable (see Tables S3–S6).

### DeepLight Accurately Identifies Physiologically Appropriate Associations Between Cognitive States and Brain Activity on Multiple Levels of Data Granularity

DeepLight's ability to correctly identify the physiological appropriate associations between cognitive states and brain activity is exemplified in Figure 5. Here, the distribution of relevance values for the four cognitive states is visualized on three different levels of data granularity of an exemplar subject (namely, the subject with the highest decoding accuracy in Figures 3A,B): First, on the level of the overall distribution of relevance values of each cognitive state of this subject (Figure 5A; incorporating an average of 47 TRs per cognitive state), then on the level of the first experiment block of each cognitive state in the first experiment run (Figure 5B; incorporating an average of 12 TRs per cognitive state) and lastly on the level of a single brain sample of each cognitive state (Figure 5C; incorporating a single TR per cognitive state).

On all three levels, DeepLight utilized the activity of a similar set of brain regions to identify each of the four cognitive states. Importantly, these regions largely overlap with those identified by the DeepLight group-level analysis (Figure 4E) as well as the results of the meta-analysis (Figure 4A).

### DeepLight's Relevance Patterns Resemble Temporo-Spatial Variability of Brain Activity Over Sequences of Single fMRI Samples

To further probe DeepLight's ability to analyze single time points, we next studied the distribution of relevance values over the course of a single experiment block (Figure 6). In particular, we plotted this distribution as a function of the fMRI sampling-time over all subjects for the first experiment block of the face and place stimulus classes in the second experiment run. We restricted this analysis to the face and place stimulus classes, as the neural networks involved in processing face and place stimuli, respectively, have been widely characterized (see, for example Haxby et al., 2001 as well as Heekeren et al., 2004). For a more detailed overview, we also created two videos for the two experiment blocks depicted in Figure 6 (Supplementary Videos 1, 2). These videos display the temporal evolution of relevance values for each fMRI sample in the original fMRI sampling time of the face (Supplementary Video 1) and place (Supplementary Video 2) experiment blocks.

In the beginning of the experiment block, DeepLight was generally uncertain which cognitive state the observed brain samples belonged to, as it assigned similar probabilities to each of the cognitive states considered (Figures 6A,B). As time progressed, however, DeepLight's certainty increased and it correctly identified the cognitive state underlying the fMRI samples. At the same time, it started assigning more relevance to the target ROIs of the face and place stimulus classes (Figures 6C–F), as indicated by the increasing F1-scores resulting from a comparison of the brain maps at each sampling time point with the results of the meta-analysis (Figures 6G,H; all brain maps were again thresholded at the 90th percentile for this comparison). Interestingly, the relevances started peaking in the target ROIs 5s after the onset of the experiment block. The temporal evolution of the relevances thereby mimics the hemodynamic response measured by the fMRI (Lindquist et al., 2009).

To further evaluate the results of this analysis, we replicated it by the use of the whole-brain lasso group-level decoding model (see section Baseline Methods and section Parameter Estimation of the Baseline Methods in Supplementary Information). In particular, we multiplied the fMRI samples of all test subjects collected at each sampling time point with the coefficient estimates of the whole-brain lasso group-level model. Subsequently, we averaged the resulting weighted fMRI samples within each sampling time point depicted in Figures 6G,H and computed an F1-score for a comparison of the resulting average brain maps with the results of the meta-analysis (as described in section Subject-Level). Interestingly, we found that the F1-scores of the whole-brain lasso analysis varied much less over the sequence of fMRI samples and were throughout lower than those of DeepLight. Thereby, indicating that the brain maps of the whole-brain lasso analysis exhibit comparably little variability over the course of an experiment block with respect to the target ROIs defined for the face and place stimulus classes.

## Discussion

Neuroimaging data have a complex temporo-spatial dependency structure that renders modeling and decoding of experimental data a challenging endeavor. With DeepLight, we propose a new data-driven framework for the analysis and interpretation of whole-brain neuroimaging data that scales well to large datasets and is mathematically non-linear, while still maintaining interpretability of the data. To decode a cognitive state, DeepLight separates a whole-brain fMRI volume into its axial slices and processes the resulting sequence of brain slices by the use of a convolutional feature extractor and LSTM. Thereby, accounting for the spatially distributed patterns of whole-brain brain activity within and across axial slices. Subsequently, DeepLight relates cognitive state and brain activity, by decomposing its decoding decisions into the contributions of the single input voxels to these decisions with the LRP method. Thus, DeepLight is able to study the associations between brain activity and cognitive state on multiple levels of data granularity, from the level of the group down to the level of single subjects, trials and time points.

To demonstrate the versatility of DeepLight, we have applied it to an openly available fMRI dataset of 100 subjects viewing images of body parts, faces, places, and tools. With these data, we have shown that the DeepLight (1) decodes the underlying cognitive states more accurately from the fMRI data than conventional means of uni- and multivariate brain decoding, (2) improves its decoding performance better with growing datasets, (3) accurately identifies the physiologically appropriate associations between cognitive states and brain activity, (4) can study these associations on multiple levels of data granularity, from the level of the group down to the level of single subjects, trials and time points, and (5) can capture the temporo-spatial variability of brain activity over sequences of single fMRI samples.

### Transferring DeepLight to Other fMRI Datasets

The DeepLight architecture used here is exemplary. Future research is needed to evaluate how the specific architectural choices for its three sub-modules (the convolutional feature extractor, LSTM unit and softmax output layer; see section DeepLight Framework) will effect its performance. In the following, we will briefly outline how the proposed architecture can be transferred to the analysis of other fMRI datasets with different spatial resolution and decoding targets. Importantly, online minimal changes are necessary in order to adapt DeepLight's architecture for the analysis of such fMRI datasets.

DeepLight first processes an fMRI volume within each axial slice, by computing a higher-level, and lower-dimensional, representation of the slices with the convolutional feature extractor. Here, the spatial sensitivity of DeepLight to the fine-grained activity differences of neighboring voxels within each slice is determined by the stride size applied by the convolution layers. The stride size indicates the distance between the application of the convolution kernels to the axial slices of the fMRI volume. Generally, a larger stride decreases DeepLight's sensitivity for fine-grained differences in the activity of neighboring voxels, as it increases the distance between the applications of the convolution kernels to the input slice. Reversely, a smaller stride size increases DeepLight's sensitivity for the fine-grained activity differences of neighboring voxels, as it decreases the distance between the applications of the convolution kernels. For example, when analyzing fMRI volumes that have a lower spatial resolution than the ones used here, containing fewer voxels per axial slice (and thereby less information about the distribution of brain activity within each slice), we would recommend to decrease the stride size for more of DeepLight's convolution layers, in order to best leverage the information contained in these voxels.

After the application of the convolutional feature extractor, DeepLight integrates the information of the resulting higher-level slice representations, by the use of a bi-directional LSTM. Here, each of the two LSTM units iterates through the entire sequence of slice representations, before forwarding its output. The proposed DeepLight architecture therefore does not require any modification in order to accommodate fMRI datasets with a different number of axial slices per volume, as it generalizes to any sequence length.

Further, the number of neurons in the softmax output layer is directly determined by the number of decoding targets considered in the data (one output neuron per decoding target). In the case of a continuous decoding target (for example, by predicting a subject's score in a cognitive test), the softmax output layer can be replaced with a linear regression layer. The LRP decomposition approach (see section Layer-Wise Relevance Propagation in the DeepLight Framework) also applies to continuous output variables (for further details on the application of the LRP approach to continuous output variables, see Bach et al., 2015 and Montavon et al., 2017).

Lastly, recent exploratory empirical work has shown that even for more complex fMRI decoding analyses, encompassing up to 400 subjects and 20 distinct cognitive states (see Thomas et al., 2019), DeepLight does not require more than 64 neurons per layer. We would therefore not recommend to increase the number of neurons further, as this will also lead to an overall increased risk of overfitting.

### Comparison to Baseline Methods

#### General Linear Model

The GLM is conceptually different from the other neuroimaging analysis approaches considered in this work. It aims to identify an association between cognitive state and brain activity, by modeling (or predicting) the time series signal of a single voxel as a linear combination of a set of experiment predictors (see section Baseline Methods). It is thereby limited in three meaningful ways that do not apply to DeepLight: First, the time series signal of a voxel is generally very noisy. The GLM treats each voxel's signal as independent of one another, thereby, not leveraging the evidence that is shared across the time series signal of multiple voxels. Second, even though the linear combination of a set of experiment predictors might be able to explain variance in the observed fMRI data, it does not necessarily provide evidence that this exact set of predictors is encoded in the neuronal response. Generally, the same linear model (in terms of its predictions) can be constructed from many different (even random) sets of predictors (for a detailed discussion of this “feature fallacy,” see Kriegeskorte and Douglas, 2018). The results of the GLM analysis thereby indicate that the measured neuronal response is highly structured and that this structure is preserved across individuals, whereas the labels assigned to its predictors might be arbitrary. Third, the performance of the GLM in predicting the response signal of a voxel is typically not evaluated on independent data, which leaves unanswered how well its results generalize to new data.

#### Searchlight Analysis

DeepLight generally outperformed the searchlight analysis in decoding the cognitive states from the fMRI data. In small datasets (here, the data of 10 or less subjects), however, the performance of the searchlight analysis was superior. In contrast to DeepLight, the searchlight analysis decodes a cognitive state from single cluster of only few voxels. Its input data, as well as the number of parameters in its decoding model, are thereby considerably smaller, leading to an overall lower risk of overfitting. Yet, this advantage comes at the cost of additional constraints that have to be considered when considering both approaches. If a cognitive state is associated with the activity of a small brain region only, the searchlight analysis will generally be more sensitive to the activity of this region than DeepLight, as it has learned a decoding model that is specific to the activity of the region. If, however, the cognitive state is not identifiable by the activity of a single brain region only, but solely in conjunction with the activity of another spatially distinct brain region, the searchlight analysis will not be able to identify this association, due to its narrow spatial focus. DeepLight, on the other hand, will generally be less sensitive to the specifics of the activity of a local brain region, but perform better in identifying a cognitive state from spatially wide-spread brain activity. When choosing between both approaches, one should therefore consider whether the assumed associations between brain activity and cognitive state specifically involve the activity of a local brain region only, or whether the cognitive state is associated with the activity of spatially distinct brain regions.

#### Whole-Brain Lasso

In contrast to DeepLight, the whole-brain lasso analysis is based on a linear decoding model. It assigns a single coefficient weight to each voxel in the brain and makes a decoding decision by computing a weighted sum over the activity of an input fMRI volume. Importantly, due to the strong regularization that is applied to the coefficients during the training, many coefficients equal 0. The resulting set of coefficients thereby resembles a brain mask, defining a set of fixed brain regions whose activity the whole-brain lasso utilizes to decode a cognitive state. DeepLight, on the other hand, utilizes a hierarchical structure of non-linear transforms of the fMRI data. It projects each fMRI volume into a more abstracted, higher-level space. This abstracted (and more flexible) view enables DeepLight to better account for the variable patterns of brain activity underlying a cognitive state (within and across individuals). This ability is exemplified in Figure 6, as well as Supplementary Videos 1, 2, where we visualize the variable patterns of brain activity that DeepLight associates with the face and place stimulus classes throughout an experiment block. The relevance patterns of DeepLight mimic the hemodynamic response and peak in the ROIs 5–10 s after the onset of the experiment block. Importantly, we find that the whole-brain lasso does not exhibit such temporo-spatial variability.

### Disentangling Temporally Distinct Associations Between Cognitive State and Brain Activity

DeepLight's ability to identify a cognitive state through variable patterns of brain activity makes it ideally suited for the analysis of the fine-grained spatial distribution of brain activity over temporal sequences of fMRI samples. For example, Hunt and Hayden (2017) recently raised the question whether the neural networks involved in reward-based decision making can be subdivided into a set of spatially distinct and temporally discrete network components, or whether the underlying networks act in parallel, with highly recurrent activity patterns. Answering this question is difficult with conventional approaches to the analysis of neuroimaging data, such as the baseline methods included in this paper. These often learn a fixed mapping between brain activity and cognitive state, by aggregating over the information provided by a sequence of fMRI samples (e.g., by estimating a single coefficient weight for each voxel from a sequence of fMRI data). The resulting brain maps thereby only indicate whether there exist spatially distinct brain regions that are associated with a cognitive state, without providing any insight whether the activity patterns are temporally discrete. While these methods can be adapted to specifically account for the temporal differences in the activity patterns of these regions (e.g., by analyzing different time points independent of one another), these adaptations often require specific hypotheses about the studied temporal differences (e.g., by needing to specify the different time points to analyze). DeepLight, on the other hand, operates purely data-driven and is thereby able to autonomously identify an association between spatially distinct patterns of brain activity and a cognitive state at temporally discrete time points.

### Integrative Analysis of Multimodal Neuroimaging Data

DeepLight is not bound to fMRI data, but can be easily extended to other neuroimaging modalities. One such complementary modality, with a higher temporal, but lower spatial resolution, is the Electroencephalography (EEG). While a plethora of analysis approaches have been proposed for the integrative analysis of EEG and fMRI data, these often incorporate restrictive assumptions to enable the integrative statistical analysis of these two data types, with clearly distinct spatial, temporal and physiological properties (for a detailed review, see Jorge et al., 2014). DeepLight, on the other hand, represents a data-driven analysis framework. By providing both, EEG and fMRI data as separate inputs to the DL model, DeepLight could learn the fine-grained temporal structure of brain activity from the EEG data, while utilizing the fMRI data to localize the spatial brain regions underlying this activity. Recently, researchers have already demonstrated the usefulness of interpretable DL methods for the analysis of EEG data (Sturm et al., 2016).

### Extending DeepLight

Lastly, we would like to highlight several possible extensions of the DeepLight approach, resulting from its flexible and modular architecture. First, DeepLight can be extended to specifically account for the temporo-spatial distribution of brain activity over sequences of fMRI samples, by the addition of another recurrent network layer. This layer would process each of the higher-level whole-brain representations resulting from the currently proposed architecture. This extension would enable DeepLight to more specifically account for the temporal distribution of brain activity. Second, DeepLight can be extended to the integrative analysis of neuroimaging data from multiple cognitive tasks and experiments. For example, by adding one neuron to the output layer for each cognitive state from each task. This extension would enable a more thorough analysis of the differences (and similarities) between the associations of cognitive state and brain activity across multiple tasks and experiments.

## Data Availability Statement

The datasets generated for this study are available on request to the corresponding author.

## Ethics Statement

The scanning protocol involving human participants were reviewed and approved by Washington University in St. Louis's Human Research Protection Office (HRPO), IRB# 201204036. No experimental activity involving the human subjects took place at the authors' institutions. The patients/participants provided their written informed consent to participate in this study. Only de-identified, publicly released data were used in this study.

## Author Contributions

AT, HH, K-RM, and WS conceived of DeepLight. AT, K-RM, and WS planned all data analyses. AT implemented all visualizations of DeepLight and the experimental procedures, performed all formal data analyses, wrote all software that was used in the data analyses and that is underlying DeepLight, and wrote the original draft of the manuscript. HH, K-RM, and WS reviewed and edited the manuscript. This work was supervised by HH, K-RM, and WS.

### Conflict of Interest

The authors declare that the research was conducted in the absence of any commercial or financial relationships that could be construed as a potential conflict of interest.

## Supplementary Material

Supplementary Video 1 DeepLight analysis of the temporo-spatial distribution of brain activity in the first experiment block of the face stimulus class in the second experiment run of the held-out test dataset. **(Right)** DeepLight's group-level brain maps for each sampling time point of the first experiment block of the face stimulus class in the second experiment run of the held-out test dataset. All brain maps are projected onto the inflated cortical surface of the FsAverage5 surface template. **(Left)** For each sampling time point, three metrics are displayed: DeepLight's softmax output prediction for each decoding target, DeepLight's average decoding accuracy, and the F1-score of a comparison of the shown group-level brain map with the results of a NeuroSynth meta-analysis for the term “face”' (for further details on the F1-score, see section Subject-Level and section NeuroSynth in Supplementary Information). Higher F1-scores generally indicate stronger similarity.

Supplementary Video 2 DeepLight analysis of the temporo-spatial distribution of brain activity in the first experiment block of the place stimulus class in the second experiment run of the held-out test dataset. **(Right)** DeepLight's group-level brain maps for each sampling time point of the first experiment block of the place stimulus class in the second experiment run of the held-out test dataset. All brain maps are projected onto the inflated cortical surface of the FsAverage5 surface template. **(Left)** For each sampling time point, three metrics are displayed: DeepLight's softmax output prediction for each decoding target, DeepLight's average decoding accuracy, and the F1-score of a comparison of the shown group-level brain map with the results of a NeuroSynth meta-analysis for the term “place”' (for further details on the F1-score, see sections Subject-Level and NeuroSynth in Supplementary Information). Higher F1-scores generally indicate stronger similarity.

## References

[B1] Abadi M. Barham P. Chen J. Chen Z. Davis A. Dean J. (2016). Tensorflow: a system for large-scale machine learning, in OSDI (Savannah, GA) Vol. 16, 265–283.

[B2] Abraham A. Pedregosa F. Eickenberg M. Gervais P. Mueller A. Kossaifi J.. (2014). Machine learning for neuroimaging with scikit-learn. Front. Neuroinform. 8:14. 10.3389/fninf.2014.00014 24600388 PMC3930868

[B3] Adolphs R. (2002). Neural systems for recognizing emotion. Curr. Opin. Neurobiol. 12, 169–177. 10.1016/S0959-4388(02)00301-X 12015233

[B4] Arras L. Montavon G. Müller K.-R. Samek W. (2017). Explaining recurrent neural network predictions in sentiment analysis, in Proceedings of the EMNLP'17 Workshop on Computational Approaches to Subjectivity, Sentiment and Social Media Analysis (WASSA) (Copenhagen: Association for Computational Linguistics), 159–168.

[B5] Bach S. Binder A. Montavon G. Klauschen F. Müller K.-R. Samek W. (2015). On pixel-wise explanations for non-linear classifier decisions by layer-wise relevance propagation. PLoS ONE 10:e0130140. 10.1371/journal.pone.0130140 26161953 PMC4498753

[B6] Barch D. M. Burgess G. C. Harms M. P. Petersen S. E. Schlaggar B. L. Corbetta M.. (2013). Function in the human connectome: task-fmri and individual differences in behavior. Neuroimage 80, 169–189. 10.1016/j.neuroimage.2013.05.033 23684877 PMC4011498

[B7] Benjamini Y. Hochberg Y. (1995). Controlling the false discovery rate: a practical and powerful approach to multiple testing. J. R. Stat. Soc. Ser. B 57, 289–300. 10.1111/j.2517-6161.1995.tb02031.x

[B8] Chang L. J. Gianaros P. J. Manuck S. B. Krishnan A. Wager T. D. (2015). A sensitive and specific neural signature for picture-induced negative affect. PLoS Biol. 13:e1002180. 10.1371/journal.pbio.1002180 26098873 PMC4476709

[B9] Cortes C. Vapnik V. (1995). Support-vector networks. Mach. Learn. 20, 273–297. 10.1007/BF00994018

[B10] Decety J. Grèzes J. (2006). The power of simulation: imagining one's own and other's behavior. Brain Res. 1079, 4–14. 10.1016/j.brainres.2005.12.115 16460715

[B11] Donahue J. Anne Hendricks L. Guadarrama S. Rohrbach M. Venugopalan S. Saenko K.. (2015). Long-term recurrent convolutional networks for visual recognition and description, in Proceedings of the IEEE Conference on Computer Vision and Pattern Recognition (Boston, MA: IEEE), 2625–2634. 10.1109/TPAMI.2016.2599174 27608449

[B12] Fischl B. (2012). Freesurfer. Neuroimage 62, 774–781. 10.1016/j.neuroimage.2012.01.021 22248573 PMC3685476

[B13] Friston K. J. Holmes A. P. Worsley K. J. Poline J.-P. Frith C. D. Frackowiak R. S. (1994). Statistical parametric maps in functional imaging: a general linear approach. Hum. Brain Mapp. 2, 189–210. 10.1002/hbm.460020402

[B14] Genovese C. R. Lazar N. A. Nichols T. (2002). Thresholding of statistical maps in functional neuroimaging using the false discovery rate. Neuroimage 15, 870–878. 10.1006/nimg.2001.1037 11906227

[B15] Glasser M. F. Sotiropoulos S. N. Wilson J. A. Coalson T. S. Fischl B. Andersson J. L.. (2013). The minimal preprocessing pipelines for the human connectome project. Neuroimage 80, 105–124. 10.1016/j.neuroimage.2013.04.127 23668970 PMC3720813

[B16] Glorot X. Bengio Y. (2010). Understanding the difficulty of training deep feedforward neural networks, in Proceedings of the 13th International Conference on Artificial Intelligence and Statistics (AISTATS) (Sardinia), 249–256.

[B17] Goodfellow I. Bengio Y. Courville A. Bengio Y. (2016). Deep Learning, Vol. 1 Cambridge: MIT press.

[B18] Goutte C. Gaussier E. (2005). A probabilistic interpretation of pre- cision, recall and f-score, with implication for evaluation, in European Conference on Information Retrieval (Santiago de Compostela: Springer), 345–359.

[B19] Gramfort A. Thirion B. Varoquaux G. (2013). Identifying predictive regions from fmri with tv-l1 prior, in 2013 International Workshop on Pattern Recognition in Neuroimaging (PRNI) (Washington, DC: IEEE), 17–20.

[B20] Grosenick L. Klingenberg B. Katovich K. Knutson B. Taylor J. E. (2013). Interpretable whole-brain prediction analysis with graphnet. Neuroimage 72, 304–321. 10.1016/j.neuroimage.2012.12.062 23298747

[B21] Haxby J. V. Gobbini M. I. Furey M. L. Ishai A. Schouten J. L. Pietrini P. (2001). Distributed and overlapping representations of faces and objects in ventral temporal cortex. Science 293, 2425–2430. 10.1126/science.1063736 11577229

[B22] Heekeren H. R. Marrett S. Bandettini P. A. Ungerleider L. G. (2004). A general mechanism for perceptual decision-making in the human brain. Nature 431:859. 10.1038/nature02966 15483614

[B23] Hochreiter S. Schmidhuber J. (1997). Long short-term memory. Neural Comput. 9, 1735–1780. 10.1162/neco.1997.9.8.1735 9377276

[B24] Hunt L. T. Hayden B. Y. (2017). A distributed, hierarchical and recurrent framework for reward-based choice. Nat. Rev. Neurosci. 18:172. 10.1038/nrn.2017.7 28209978 PMC5621622

[B25] Jang H. Plis S. M. Calhoun V. D. Lee J.-H. (2017). Task-specific feature extraction and classification of fmri volumes using a deep neural network initialized with a deep belief network: evaluation using sensorimotor tasks. Neuroimage 145, 314–328. 10.1016/j.neuroimage.2016.04.003 27079534 PMC5064875

[B26] Jorge J. Van der Zwaag W. Figueiredo P. (2014). Eeg–fmri integration for the study of human brain function. Neuroimage 102, 24–34. 10.1016/j.neuroimage.2013.05.114 23732883

[B27] Kriegeskorte N. Douglas P. K. (2018). Interpreting encoding and decoding models. Curr. Opin. Neurobiol. 55, 167–179. 10.1016/j.conb.2019.04.002 31039527 PMC6705607

[B28] Kriegeskorte N. Goebel R. Bandettini P. (2006). Information-based functional brain mapping. Proc. Natl. Acad. Sci. U.S.A. 103, 3863–3868. 10.1073/pnas.0600244103 16537458 PMC1383651

[B29] Lapuschkin S. Binder A. Montavon G. Müller K.-R. Samek W. (2016). The layer-wise relevance propagation toolbox for artificial neural networks. J. Mach. Learn. Res. 17, 1–5. Available online at: http://www.jmlr.org/papers/v17/15-618.html

[B30] Lapuschkin S. Wäldchen S. Binder A. Montavon G. Samek W. Müller K.-R. (2019). Unmasking clever hans predictors and assessing what machines really learn. Nat. Commun. 10:1096. 10.1038/s41467-019-08987-4 30858366 PMC6411769

[B31] LeCun Y. Bengio Y. (1995). Convolutional networks for images, speech, and time series, in The Handbook of Brain Theory and Neural Networks, eds Arbib M. A. (Cambridge, MA: MIT Press), 3361.

[B32] LeCun Y. Bengio Y. Hinton G. (2015). Deep learning. Nature 521:436. 10.1038/nature14539 26017442

[B33] Lindquist M. A. Loh J. M. Atlas L. Y. Wager T. D. (2009). Modeling the hemodynamic response function in fmri: efficiency, bias and mis-modeling. Neuroimage 45, S187–S198. 10.1016/j.neuroimage.2008.10.065 19084070 PMC3318970

[B34] Marban A. Srinivasan V. Samek W. Fernandez J. Casals A. (2019). A recurrent convolutional neural network approach for sensorless force estimation in robotic surgery. Biomed. Signal Process. Control. 50, 134–150. 10.1016/j.bspc.2019.01.011

[B35] McIntosh A. R. Lobaugh N. J. (2004). Partial least squares analysis of neuroimaging data: applications and advances. Neuroimage 23, S250–S263. 10.1016/j.neuroimage.2004.07.020 15501095

[B36] McLaughlin N. Martinez del Rincon J. Miller P. (2016). Recurrent convolutional network for video-based person re-identification, in Proceedings of the IEEE Conference on Computer Vision and Pattern Recognition (Las Vegas, NV: IEEE), 1325–1334.

[B37] Mensch A. Mairal J. Thirion B. Varoquaux G. (2018). Extracting universal representations of cognition across brain-imaging studies. arXiv preprint arXiv:1809.06035.

[B38] Michel V. Gramfort A. Varoquaux G. Eger E. Thirion B. (2011). Total variation regularization for fMRI-based prediction of behavior. IEEE Trans. Med. Imaging 30, 1328–1340. 10.1109/TMI.2011.2113378 21317080 PMC3336110

[B39] Montavon G. Bach S. Binder A. Samek W. Müller K.-R. (2017). Explaining nonlinear classification decisions with deep taylor decomposition. Pattern Recognit. 65, 211–222. 10.1016/j.patcog.2016.11.008

[B40] Montavon G. Samek W. Müller K.-R. (2018). Methods for interpreting and understanding deep neural networks. Digit. Signal Process. 73, 1–15. 10.1016/j.dsp.2017.10.011

[B41] Müller K.-R. Mika S. Rätsch G. Tsuda K. Schölkopf B. (2001). An introduction to kernel-based learning algorithms. IEEE Transac. Neural Netw. 12, 181–201. 10.1109/72.914517 18244377

[B42] Naselaris T. Kay K. N. Nishimoto S. Gallant J. L. (2011). Encoding and decoding in fmri. Neuroimage 56, 400–410. 10.1016/j.neuroimage.2010.07.073 20691790 PMC3037423

[B43] Nie D. Zhang H. Adeli E. Liu L. Shen D. (2016). 3d deep learning for multi- modal imaging-guided survival time prediction of brain tumor patients, in International Conference on Medical Image Computing and Computer-Assisted Intervention (Cham: Springer), 212–220. 10.1007/978-3-319-46723-8_25 PMC5278791 28149967

[B44] Olson I. R. Plotzker A. Ezzyat Y. (2007). The enigmatic temporal pole: a review of findings on social and emotional processing. Brain 130, 1718–1731. 10.1093/brain/awm052 17392317

[B45] Pascanu R. Mikolov T. Bengio Y. (2013). On the difficulty of training recurrent neural networks, in International Conference on Machine Learning (Atlanta, GA), 1310–1318.

[B46] Petrov D. Kuznetsov B. A. van Erp T. G. Turner J. A. Schmaal L. Veltman D. (2018). Deep learning for quality control of subcortical brain 3d shape models, in International Workshop on Shape in Medical Imaging, ShapeMI 2018 held in conjunction with 21st International Conference on Medical Image Computing and Computer Assisted Intervention, MICCAI 2018 (Granada: Springer Verlag, 268–276.

[B47] Plis S. M. Hjelm D. R. Salakhutdinov R. Allen E. A. Bockholt H. J. Long J. D.. (2014). Deep learning for neuroimaging: a validation study. Front. Neurosci. 8:229. 10.3389/fnins.2014.00229 25191215 PMC4138493

[B48] Poldrack R. A. Barch D. M. Mitchell J. Wager T. Wagner A. D. Devlin J. T.. (2013). Toward open sharing of task-based fmri data: the openfmri project. Front. Neuroinform. 7:12. 10.3389/fninf.2013.00012 23847528 PMC3703526

[B49] Rumelhart D. E. Hinton G. E. Williams R. J. (1986). Learning represen-tations by back-propagating errors. Nature 323:533 10.1038/323533a0

[B50] Ryali S. Supekar K. Abrams D. A. Menon V. (2010). Sparse logistic regression for whole-brain classification of fmri data. Neuroimage 51, 752–764. 10.1016/j.neuroimage.2010.02.040 20188193 PMC2856747

[B51] Samek W. Wiegand T. Müller K.-R. (2018). Explainable artificial intelligence: understanding, visualizing and interpreting deep learning models. ITU J. ICT Discov. 1, 39–48.

[B52] Sarraf S. Tofighi G. (2016). Classification of alzheimer's disease using fmri data and deep learning convolutional neural networks. arXiv preprint arXiv:1603.08631.

[B53] Schölkopf B. Smola A. J. (2002). Learning With Kernels: Support Vector Machines, Regularization, Optimization, and Beyond. Cambridge, MA: MIT press.

[B54] Srivastava N. Hinton G. Krizhevsky A. Sutskever I. Salakhutdinov R. (2014). Dropout: a simple way to prevent neural networks from overfitting. J. Mach. Learn. Res. 15, 1929–1958. Available online at: http://jmlr.org/papers/v15/srivastava14a.html

[B55] Sturm I. Lapuschkin S. Samek W. Müller K.-R. (2016). Interpretable deep neural networks for single-trial eeg classification. J. Neurosci. Methods. 274, 141–145. 10.1016/j.jneumeth.2016.10.008 27746229

[B56] Suk H.-I. Lee S.-W. Shen D. the Alzheimers Disease Neuroimaging Initiative. (2014). Hierarchical feature representation and multimodal fusion with deep learning for ad/mci diagnosis. Neuroimage 101, 569–582. 10.1016/j.neuroimage.2014.06.077 25042445 PMC4165842

[B57] Thomas A. W. Heekeren H. R. Mller K.-R. Samek W. (2018). Analyzing neuroimaging data through recurrent deep learning models. arXiv preprint arXiv:1810.09945. 10.3389/fnins.2019.01321 PMC6914836 31920491

[B58] Thomas A. W. Müller K. R. Samek W. (2019). Deep transfer learning for whole-brain FMRI analyses, in OR 2.0 Context-Aware Operating Theaters and Machine Learning in Clinical Neuroimaging, eds Zhou L. Sarikaya D. Kia S. M. Speidel S. Malpani A. Hashimoto D. Habes M. Löfstedt T. Ritter K. Wang H. (Cham: Springer, 59–67.

[B59] Tibshirani R. (1996). Regression shrinkage and selection via the lasso. J. R. Stat. Soc. Ser. B 58, 267–288. 10.1111/j.2517-6161.1996.tb02080.x

[B60] Tikhonov A. N. (1943). On the stability of inverse problems. Dokl. Akad. Nauk SSSR 39, 195–198.

[B61] Uğurbil K. Xu J. Auerbach E. J. Moeller S. Vu A. T. Duarte-Carvajalino J. M.. (2013). Pushing spatial and temporal resolution for functional and diffusion mri in the human connectome project. Neuroimage 80, 80–104. 10.1016/j.neuroimage.2013.05.012 23702417 PMC3740184

[B62] Van Essen D. C. Smith S. M. Barch D. M. Behrens T. E. Yacoub E. Ugurbil K.. (2013). The wu-minn human connectome project: an overview. Neuroimage 80, 62–79. 10.1016/j.neuroimage.2013.05.041 23684880 PMC3724347

[B63] Wager T. D. Atlas L. Y. Lindquist M. A. Roy M. Woo C.-W. Kross E. (2013). An fmri-based neurologic signature of physical pain. N. Eng. J. Med. 368, 1388–1397. 10.1056/NEJMoa1204471 23574118 PMC3691100

[B64] Yarkoni T. Poldrack R. A. Nichols T. E. Van Essen D. C. Wager T. D. (2011). Large-scale automated synthesis of human functional neuroimaging data. Nat. Methods 8:665. 10.1038/nmeth.1635 21706013 PMC3146590

[B65] Yousefnezhad M. Zhang D. (2018). Anatomical pattern analysis for decoding visual stimuli in human brains. Cognit. Comput. 10, 284–295. 10.1007/s12559-017-9518-9

