# Supplementary material for: Analyzing Neuroimaging Data Through Recurrent Deep Learning Models
Source: Front Neurosci. 2019 Dec 10;13:1321. doi: 10.3389/fnins.2019.01321 (PMC6914836; doi:10.3389/fnins.2019.01321)
Supplement: Supplementary file 1 [file Data_Sheet_1.pdf]

Supplementary Information

1. Parameter estimation of the baseline methods

Throughout the following, we will describe the procedures that we used to estimate a set of brain maps for each cognitive state with each of the baseline analysis approaches. Importantly, due to the diverse statistical nature of the different analysis approaches (see section Baseline methods of the main text), the values of their brain maps have different scales and interpretations.

As our analyses are sub-divided into a separate analysis on the subject- and group-level (containing the data of an individual subject or the data of the entire group of subjects), we will also divide the following section according to the subject- and group-level. The data of many subjects (with approximately 1GB per subject) can easily exceed the working memory capacities of a regular working station. For this reason, we adapted the parameter estimation procedures for the searchlight analysis and whole-brain lasso, when switching from the subject- to the group-level.

1.1 Subject-level

General Linear Model

Our GLM analyses included one predictor for each of the four cognitive states in the design matrix (each representing a box-car function for the occurrence of a cognitive state; for methodological details on the GLM, see section Baseline methods of the main text). We convolved these predictors with a canonical hemodynamic response function (HRF; [Lindquist et al., 2009]), as implemented in NiPy 0.4.1 [Gorgolewski et al., 2011], to generate the model predictors. We added temporal derivative terms derived from each predictor, an intercept and an indicator of the experiment run to the design matrix, which we all treated as confounds of no interest. The derivative terms were computed by the use of the cosine drift model as implemented in NiPy 0.4.1 [Gorgolewski et al., 2011]. All β-coefficients and error terms of the GLM analysis were estimated by the use of a first-level autoregressive model, as implemented in NiPy 0.4.1 [Gorgolewski et al., 2011]. To generate a set of subject-level brain maps, we computed a linear first-level contrast within the data of each individual subject (representing a linear contrast between one of the cognitive states and all others). The resulting brain maps indicate the estimated Z-values of these contrasts.

Searchlight analysis

To obtain a set of subject-level brain maps for each cognitive state with the searchlight analysis, we trained searchlight in a one-vs-rest procedure. Here, one SVM classifier is trained at each location in the brain to distinguish each cognitive state from all others. A decoding decision is then made according to the classifier with the most certainty that the sample belongs to its respective cognitive state. We first trained the searchlight analysis within the data of the first experiment run of a subject (see section Experiment paradigm of the main text) and subsequently predicted the cognitive states underlying the data of the second experiment run. The resulting brain maps indicate the decoding accuracies achieved by each of these SVM classifiers in the second experiment run at each searchlight location in the brain.

Whole-brain Least Absolute Shrinkage Logistic Regression

Similarly, we also trained the whole-brain lasso in a one-vs-rest procedure. To determine the magnitude of the regularization parameter λ, we additionally applied a grid search. First, we split the full training

data of a subject, containing the data of the first experiment run (see section Experiment paradigm of the main text), into the eight experiment blocks of this run (two per cognitive state). We then separated these blocks into a new training dataset (containing the first experiment block of each cognitive state) and a new validation dataset (containing the second experiment block of each cognitive state). Subsequently, for each λ-value of the parameter grid, we fit the whole-brain lasso to the data of the newly formed training dataset and evaluated its performance on the new validation data. Importantly, we utilized a logistic model implementation of the scikit-learn python library [Abraham et al., 2014]. Here, the regularization parameter (C) is implemented inversely to the regularization strength λ (with lower values indicating stronger regularization; see eq. 3). With this procedure, we evaluated a grid of 100 logarithmically spaced C-values between 1e-6 and 100. From these values we then selected the C-parameter for the subject that achieved the highest decoding accuracy in the new validation dataset (for an overview of the selected subject C-parameters, see Supplementary Information Table 1). Subsequently, we used the selected C-value to fit the whole-brain lasso to the full training data of the subject (containing the entire data of the first experiment run). The resulting brain maps of the whole-brain lasso show the coefficient estimates of each of these one-vs-rest logistic models.

1.2 Group-level

General Linear Model

To generate a set of group-level brain maps with the GLM, we computed a second-level GLM contrast by the use of the standard two-stage procedure for a random-effects group-level analysis, as proposed by Holmes & Friston [Holmes and Friston, 1998]. Here, the subject-level regression coefficients β (see section Baseline methods of the main text) are treated as random effects in a second-level linear contrast analysis, where the distribution of first-level β-contrasts is assessed by the use of a one-sample t-test (again, contrasts were computed between each cognitive state and all others). The resulting group-level brain maps show the t-values resulting from this test.

Searchlight analysis

For the group-level searchlight analysis, we trained and evaluated the searchlight on the β-coefficient maps of a first-level GLM analysis of each individual subject (resulting in one β-coefficient map per subject and cognitive state; see Supplementary Information section 1.1). This is a common approach for group-level predictions with the searchlight analysis and is widely applied in the neuroscience literature [Helfinstein et al., 2014, Reverberi et al., 2018, Schuck et al., 2016, Weygandt et al., 2012]. First, we trained the searchlight analysis in a one-vs-rest procedure on the subject-level β-coefficient maps of the training dataset. Subsequently, we used each of the trained searchlight classifiers to decode the cognitive states underlying each subject-level β-coefficient map in the test data. The resulting group-level brain maps represent the decoding accuracies achieved by each of these searchlight classifiers in the test data at each location in the brain.

Whole-brain Least Absolute Shrinkage Logistic Regression

On the group-level, we trained the whole-brain lasso in a stochastic gradient descent learning procedure [Kiefer et al., 1952]. Here, the regularized logistic model (see eq. 3) is fit iteratively to subsets of the full training data. At each iteration, the gradient of the loss function is estimated and the model's parameters are updated accordingly. To determine the strength of the regularization parameter λ, we again applied a grid search procedure. For each value of the λ-grid, we trained the whole-brain lasso over 25 epochs. In each epoch, we randomly selected the fMRI data of five subjects from the training dataset. We then randomly drew 50 batches, each containing 50 randomly drawn TRs, and updated the whole-brain lasso parameters iteratively for each batch. After completing the 25 epochs, we evaluated the decoding performance of the whole-brain lasso on the full test dataset. Overall, we evaluated 20 different λ-parameters in this grid-search and selected the λ-value achieving the highest decoding accuracy in the test dataset (λ=0.0001; for an overview of the evaluated λ-values and resulting decoding accuracies, see Supplementary Information Table S2). We then used the selected λ-parameter to train the whole-brain

lasso in the same stochastic gradient procedure described before. This time, however, spanning 200 training epochs. The group-level brain maps of the whole-brain lasso represent the resulting one-vs-rest logistic model coefficients.

2. NeuroSynth

The goal of NeuroSynth [Yarkoni et al., 2011] is to provide an automated meta-analysis database relating cognitive states and brain activity. For specific cognitive states (i.e., “pain”), the NeuroSynth database incorporates a large record of neuroimaging studies that used this term at a high frequency (>1 in 1000 words) in the article text. For these studies, the database includes the activation coordinates from all tables that are reported in these studies, producing a large set of *term-to-activation* mappings. Based on these mappings, NeuroSynth provides two types of tests: a *uniformity test*, indicating whether the probability that an article reports a specific brain activation is different, if it includes a specific term, compared to when brain activation would be distributed uniformly throughout gray matter and an *association test*, indicating whether the probability that a research article reports a specific brain activation is different, if it includes a specific term, compared to when it does not.

For our analyses, we used the latter, association test, as recommended by the NeuroSynth authors [Yarkoni et al., 2011], and extracted the thresholded ($P \leq 0.01$, voxel-wise false discovery rate corrected [Benjamini and Hochberg, 1995, Genovese et al., 2002]) brain maps for the four stimulus classes (indicated by the terms "body”, "face”, "place” and "tools”). These maps indicate a Z-value for the previously described association test at each coordinate in the MNI-space.

3. F1-score

The F1-score for a binary classifier and a given dataset is defined as the harmonic mean of its precision and recall:

$$F_1 = 2\frac{precision \cdot recall}{precision + recall} \tag{1}$$

Here, the classifier’s precision is defined as the fraction of samples in the dataset that it correctly classified as positives, given the total number of samples that it classified as positive in the dataset, whereas its recall describes the fraction of samples that it correctly classified as positive, given the overall number of positive samples in the dataset.

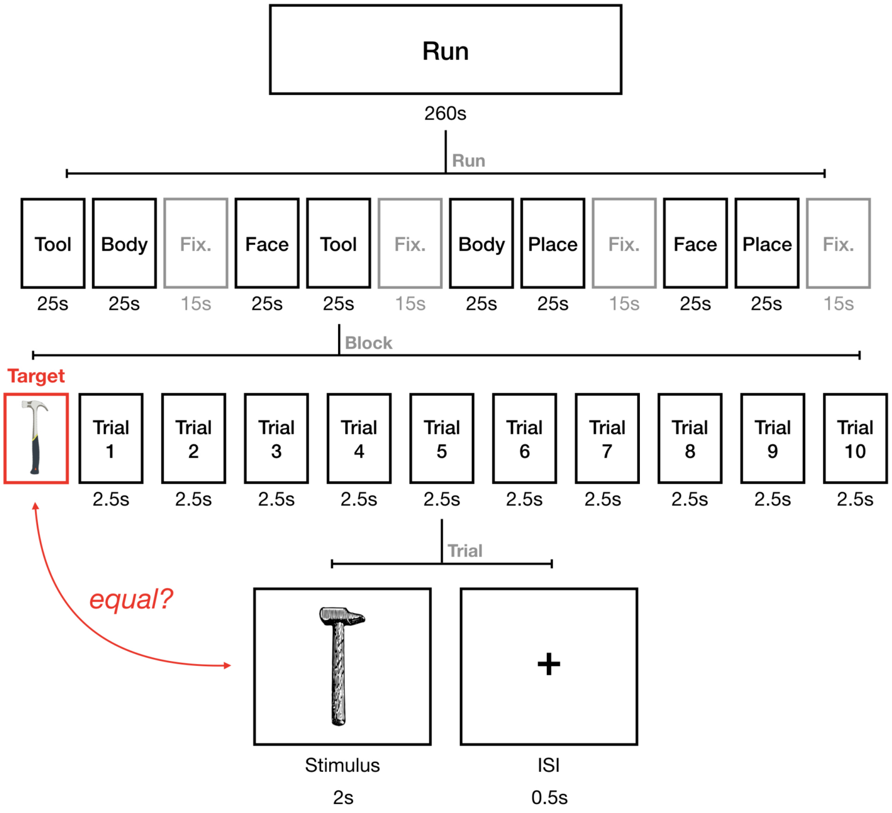

Figure S1: Experimental Paradigm. 100 subjects completed two experiment runs in the fMRI. Each run consisted of eight task and four fixation blocks. The four stimulus types (body, face, place, tool) were presented in separate blocks. Each task block consisted of 10 trials. In each trial, a stimulus was presented for 2s, followed by a 500ms interstimulus interval. Subjects performed an N-back task, in which they were asked to respond "target" when the currently presented stimulus was the same as a target stimulus. The target was either presented at the beginning of the block (0-back) or subjects were asked to indicate whether the current stimulus was the same as the stimulus two back (2-back). Half of the blocks used a 2-back and the other half a 0-back condition. For illustrative purposes only the 0-back task is depicted.

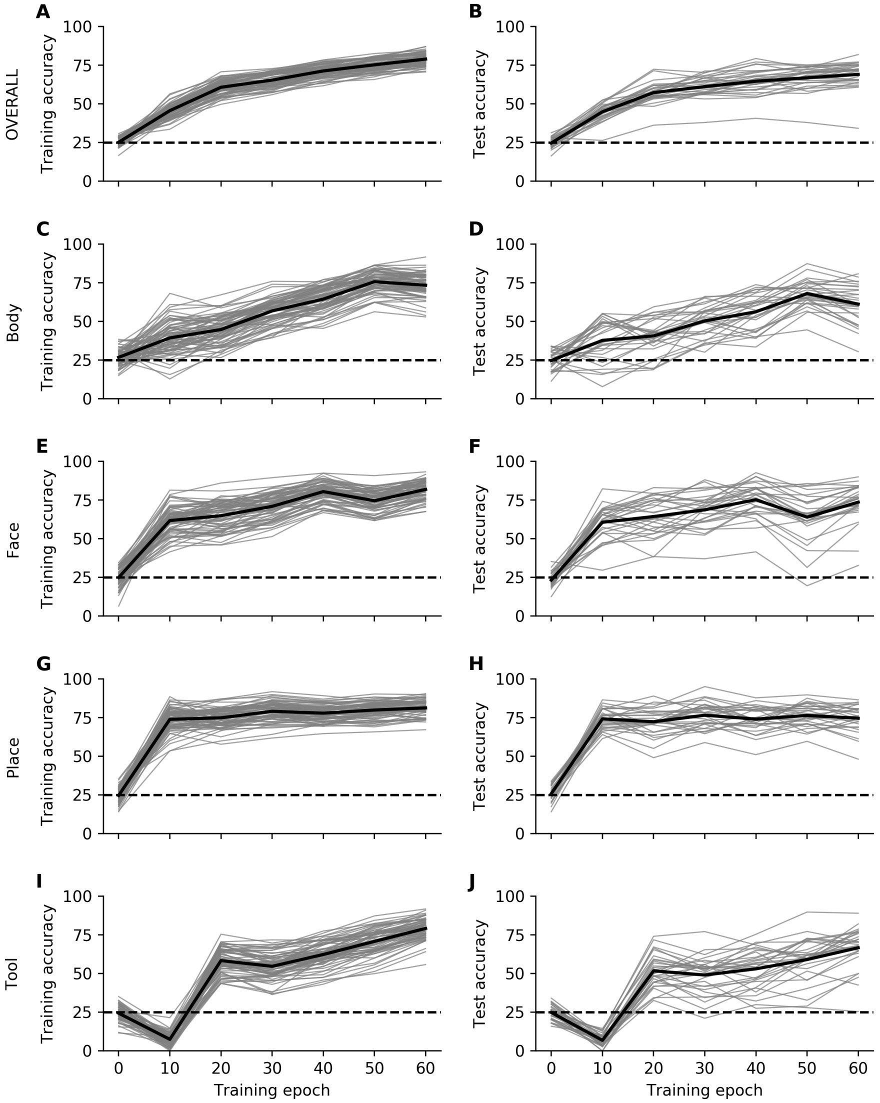

Figure S2: DeepLight's decoding accuracy as a function of the training epochs. A-B: Overall decoding accuracy achieved in the training (A) and test (B) data. Thick black lines indicate the grand average, whereas thin grey lines indicate individual subjects. Decoding accuracy in the training and test data for the body (C-D), face (E-F), place (G-H) and tool (I-J) stimulus classes. An epoch is defined as a full iteration over the training data. We define decoding accuracy as the fraction of samples in the data that were classified correctly. For further details on DeepLight's training, see the Methods section of the main text.

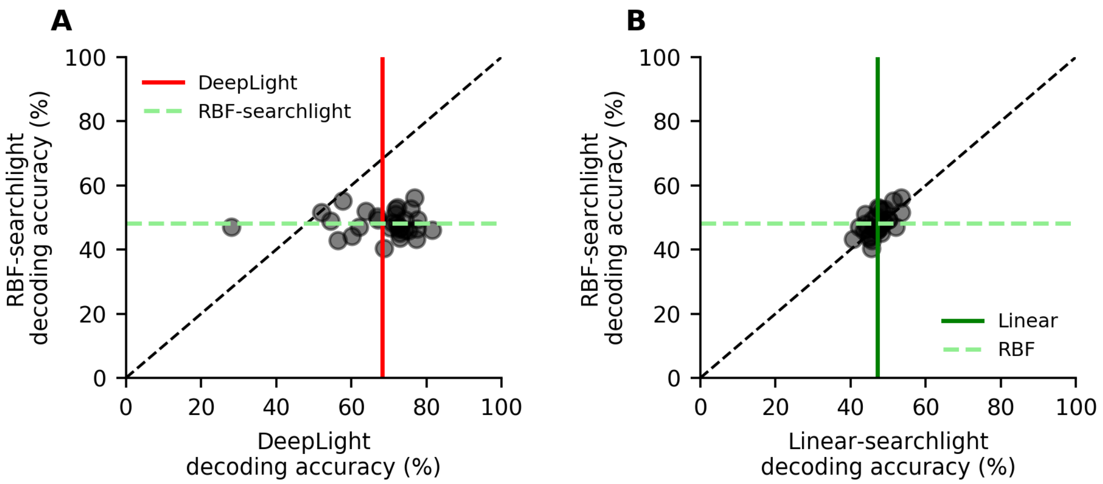

Figure S3: Out of sample decoding performance comparison of the searchlight analysis with a linear-kernel SVM and a non-linear radial basis function (RBF) kernel SVM. We trained each of the two searchlight variants on the data of the first experiment run of a subject, before predicting the cognitive state for each TR of the second experiment run (for details on the estimation procedures, see Supplementary Information section 1). We performed this prediction exercise only within the data of the subjects in the held-out test dataset. We fixed the searchlight radius to 5.6mm, while we set γ parameter of the RBF-kernel to 1 across all subjects. A: Decoding performance comparison of the RBF-kernel SVM with DeepLight. B: Decoding performance comparison of the RBF-kernel SVM with the linear-kernel SVM. Black points indicate average decoding accuracies of individual subjects. Colored lines indicate averages across subjects. For an overview of the statistical results of the comparison, see the Results section of the main text.

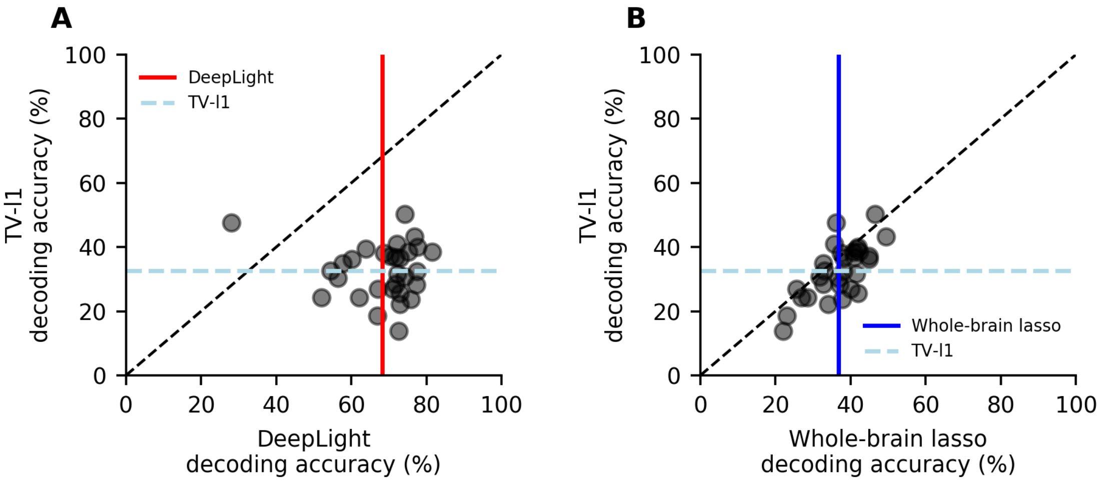

Figure S4: Out of sample decoding performance comparison of the whole-brain lasso to that of its TV-l1 extension. TV-l1 extends the logistic regression model of the whole-brain lasso, by combining the l1-penalty of the whole-brain lasso (see section Baseline methods of the main text) with an additional Total-Variation (TV) penalty to better account for the spatial dependency structure of fMRI data (for details on the TV-l1 approach, see [Gramfort et al., 2013]). The trade-off between both penalty terms is determined by a mixing constant (the l1-ratio, bounded between [0,1]), which determines the ratio at which both penalty terms are mixed during regularization (with larger l1-ratio values indicating stronger l1-regularization). To estimate the parameters of both decoding approaches, we first trained both decoding models on the data of the first experiment run of a subject (for details on the subject-level estimation procedures of the whole-brain lasso, see Supplementary section 1). Subsequently, we used the trained decoding models to predict the cognitive state underlying each fMRI volume of the second experiment run of the same subject. We performed this prediction exercise only within the data of the 30 subjects in the held-out test dataset (see section FMRI data acquisition & preprocessing of the main text). To determine the best fitting l1-ratio of TV-l1 for each subject, we evaluated five different l1-ratios (namely, 0.1, 0.3, 0.5, 0.7, 0.9) in a 3-fold cross-validation procedure within the data of the first experiment run of a subject. We then selected the l1-ratio achieving the highest decoding accuracy across the three folds. The overall strength of the regularization (as determined by the λ-parameter, see section Baseline methods of the main text), was determined through the default grid-search procedure implemented by the Nilearn toolbox [Abraham et al., 2014]. Here, an additional grid of 10 distinct λ-values is evaluated in the same 3-fold cross-validation procedure described before. A: Decoding performance comparison of TV-l1 with DeepLight. B: Decoding performance comparison of TV-l1 with the whole-brain lasso. Each point represents an individual subject. Colored lines indicate averages across subjects. For an overview of the statistical results of the comparison, see the Results section of the main text.

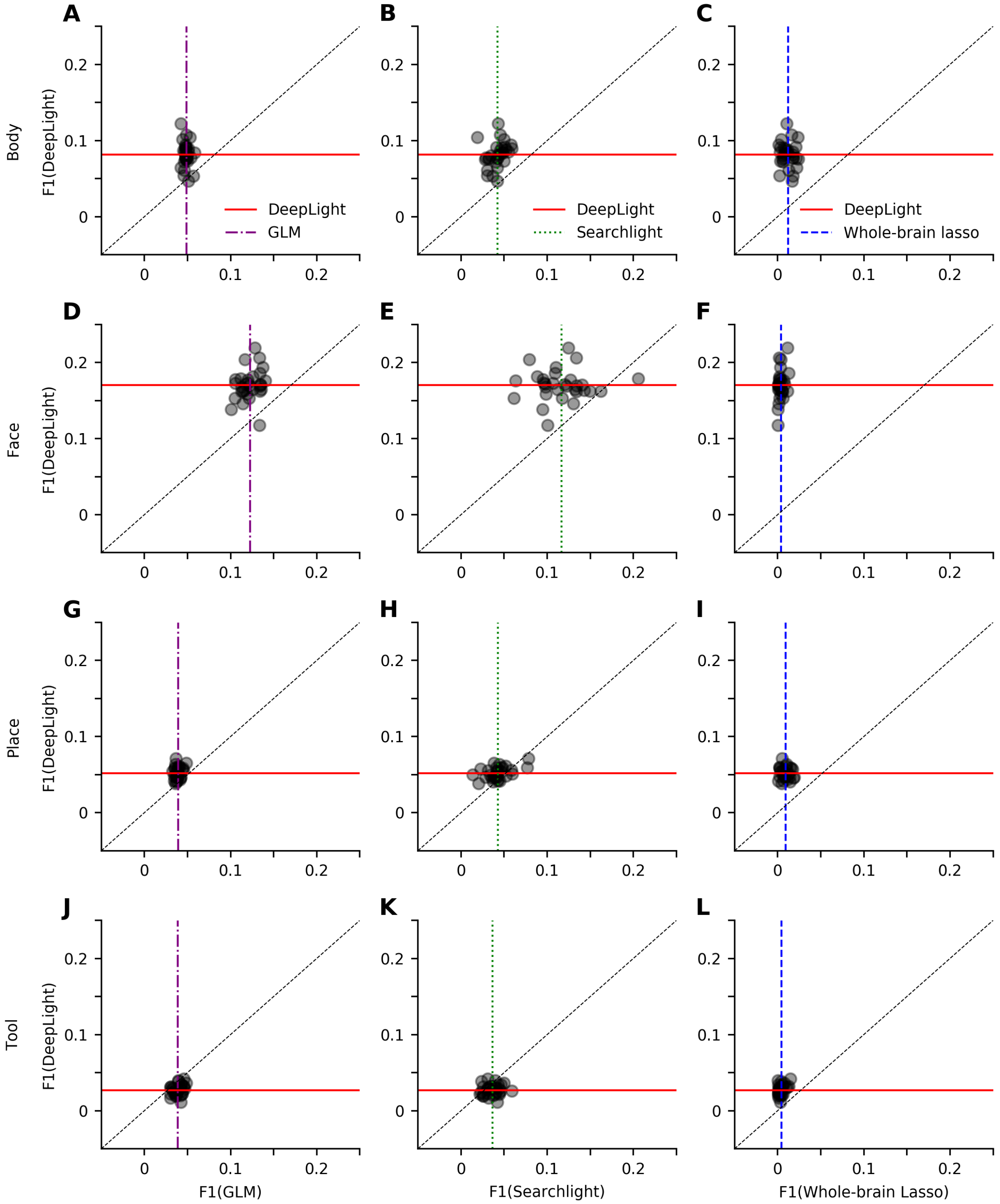

Figure S5: Comparison of the subject-level F1-scores of DeepLight to the GLM (A,D,G,J), searchlight analysis (B,E,H,K) and whole-brain lasso (C,F,I,L). The brain maps of DeepLight, the searchlight analysis and whole-brain lasso were thresholded at the 90th percentile of the values within each map, whereas the GLM brain maps were thresholded at a P-value of 0.005 (uncorrected). Points indicate the F1-scores of individual subjects. Colored lines indicate average F1-scores across subjects. For an overview of the statistical results of the comparison, see the Results section of the main text.

Test Subject	C	Decoding Accuracy
1	18.74	33%
2	0.26	33%
3	0.09	42%
4	0.66	42%
5	0.22	47%
6	0.26	49%
7	0.79	37%
8	22.57	42%
9	12.92	26%
10	83.02	40%
11	39.44	27%
12	22.57	37%
13	39.44	32%
14	0.26	42%
15	0.79	38%
16	3.51	38%
17	3.51	41%
18	0.26	22%
19	0.15	36%
20	27.19	38%
21	32.75	45%
22	7.39	34%
23	6.14	41%
24	7.39	45%
25	0.66	29%
26	4.23	38%
27	2.92	33%
28	47.51	42%
29	12.92	36%
30	1.67	23%

Table S1: Selected regularization strength parameters for the subject-level whole-brain lasso analysis (for details on the underlying subject-level grid search procedure, see Supplementary Information section 1.1). For each subject, the selected regularization parameter (C) and resulting decoding accuracy are presented.

λ	Decoding Accuracy
1e-7	46.62%
1e-6	46.40%
1e-5	47.82%
**0.0001**	**48.11%**
0.0002	47.24%
0.0003	47.42%
0.0005	46.11%
0.0008	47.03%
0.001	45.53%
0.002	45.58%
0.004	44.98%
0.007	44.86%
0.01	42.89%
0.02	40.69%
0.03	37.14%
0.06	35.48%
0.1	26.57%
0.2	25.13%
0.3	25.00%
0.5	25.09%

Table S2: λ parameters of the group-level whole-brain lasso anlaysis that were evaluated in the grid search procedure (for details on the group-level grid search, see Supplementary Information section 1.2). The respective decoding accuracy in the test dataset is given for each λ value.

P	Percentile	GLM	Searchlight	Whole-brain lasso
0.05	85	t(29)=8.54, p<0.0001	t(29)=11.99, p<0.0001	t(29)=21.64, p<0.0001
0.05	90	t(29)=10.46, p<0.0001	t(29)=13.26, p<0.0001	t(29)=20.93, p<0.0001
0.05	95	t(29)=11.43, p<0.0001	t(29)=14.04, p<0.0001	t(29)=18.41, p<0.0001
0.005	85	t(29)=8.54, p<0.0001	t(29)=11.99, p<0.0001	t(29)=21.61, p<0.0001
0.005	90	t(29)=10.46, p<0.0001	t(29)=13.26, p<0.0001	t(29)=20.93, p<0.0001
0.005	95	t(29)=11.43, p<0.0001	t(29)=14.04, p<0.0001	t(29)=18.41, p<0.0001
0.0005	85	t(29)=8.54, p<0.0001	t(29)=11.99, p<0.0001	t(29)=21.61, p<0.0001
0.0005	90	t(29)=10.45, p<0.0001	t(29)=13.26, p<0.0001	t(29)=20.93, p<0.0001
0.0005	95	t(29)=11.42, p<0.0001	t(29)=14.04, p<0.0001	t(29)=18.41, p<0.0001
0.000 05	85	t(29)=8.54, p<0.0001	t(29)=11.99, p<0.0001	t(29)=21.61, p<0.0001
0.000 05	90	t(29)=10.46, p<0.0001	t(29)=13.26, p<0.0001	t(29)=20.93, p<0.0001
0.000 05	95	t(29)=11.42, p<0.0001	t(29)=14.04, p<0.0001	t(29)=18.41, p<0.0001

Table S3: Results of a two-sided t-test of the difference between the subject-level F1-scores for the body stimulus class of each of the competing analysis approaches with the subject-level F1-scores of DeepLight. The t-test comparison was repeated for each combination of percentile- and P-threshold (for details on the F1-score comparison procedure, see the Results section of the main text and Supplementary Information section 3). T-tests were performed using a Bonferroni adjusted alpha level of 0.0014 (0.05/36). Bold font indicates t-tests with p-values greater than 0.0014.

P	Percentile	GLM	Searchlight	Whole-brain lasso
0.05	85	t(29)=16.26, p<0.0001	t(29)=9.23, p<0.0001	t(29)=61.12, p<0.0001
0.05	90	t(29)=13.04, p<0.0001	t(29)=8.57, p<0.0001	t(29)=48.32, p<0.0001
0.05	95	t(29)=4.97, p=0.00027	t(29)=7.01, p<0.0001	t(29)=33.92, p<0.0001
0.005	85	t(29)=16.26, p<0.0001	t(29)=9.23, p<0.0001	t(29)=61.12, p<0.0001
0.005	90	t(29)=13.04, p<0.0001	t(29)=8.57, p<0.0001	t(29)=48.32, p<0.0001
0.005	95	t(29)=4.97, p=0.00027	t(29)=7.01, p<0.0001	t(29)=33.92, p<0.0001
0.0005	85	t(29)=16.26, p<0.0001	t(29)=9.23, p<0.0001	t(29)=61.12, p<0.0001
0.0005	90	t(29)=13.04, p<0.0001	t(29)=8.57, p<0.0001	t(29)=48.32, p<0.0001
0.0005	95	t(29)=4.97, p=0.00027	t(29)=7.01, p<0.0001	t(29)=33.92, p<0.0001
0.000 05	85	t(29)=16.26, p<0.0001	t(29)=9.23, p<0.0001	t(29)=61.12, p<0.0001
0.000 05	90	t(29)=13.04, p<0.0001	t(29)=8.57, p<0.0001	t(29)=48.32, p<0.0001
0.000 05	95	t(29)=4.97, p=0.00027	t(29)=7.01, p<0.0001	t(29)=33.92, p<0.0001

Table S4: Results of a two-sided t-test of the difference between the subject-level F1-scores for the face stimulus class of each of the competing analysis approaches with the subject-level F1-scores of DeepLight. The t-test comparison was repeated for each combination of percentile- and P-threshold (for details on the F1-score comparison procedure, see the Results section of the main text and Supplementary Information section 3). T-tests were performed using a Bonferroni adjusted alpha level of 0.0014 (0.05/36). Bold font indicates t-tests with p-values greater than 0.0014.

P	Percentile	GLM	Searchlight	Whole-brain lasso
0.05	85	t(29)=5.02, p=0.00024	**t(29)=1.49, p=0.15**	t(29)=20.56, p<0.0001
0.05	90	t(29)=9.26, p<0.0001	t(29)=4.25, p=0.0002	t(29)=22.43, p<0.0001
0.05	95	t(29)=11.87, p<0.0001	t(29)=7.63, p<0.0001	t(29)=22.38, p<0.0001
0.005	85	t(29)=5.02, p=0.00024	**t(29)=1.49, p=0.15**	t(29)=20.56, p<0.0001
0.005	90	t(29)=9.26, p<0.0001	t(29)=4.25, p=0.0002	t(29)=22.43, p<0.0001
0.005	95	t(29)=11.87, p<0.0001	t(29)=7.63, p<0.0001	t(29)=22.38, p<0.0001
0.0005	85	t(29)=5.02, p=0.00024	**t(29)=1.49, p=0.15**	t(29)=20.56, p<0.0001
0.0005	90	t(29)=9.26, p<0.0001	t(29)=4.25, p=0.0002	t(29)=22.43, p<0.0001
0.0005	95	t(29)=11.87, p<0.0001	t(29)=7.63, p<0.0001	t(29)=22.38, p<0.0001
0.000 05	85	t(29)=5.02, p=0.00024	**t(29)=1.49, p=0.15**	t(29)=20.56, p<0.0001
0.000 05	90	t(29)=9.26, p<0.0001	t(29)=4.25, p=0.0002	t(29)=22.43, p<0.0001
0.000 05	95	t(29)=11.87, p<0.0001	t(29)=7.63, p<0.0001	t(29)=22.38, p<0.0001

Table S5: Results of a two-sided t-test of the difference between the subject-level F1-scores for the place stimulus class of each of the competing analysis approaches with the subject-level F1-scores of DeepLight. The t-test comparison was repeated for each combination of percentile- and P-threshold (for details on the F1-score comparison procedure, see the Results section of the main text and Supplementary Information section 3). T-tests were performed using a Bonferroni adjusted alpha level of 0.0014 (0.05/36). Bold font indicates t-tests with p-values greater than 0.0014.

P	Percentile	GLM	Searchlight	Whole-brain lasso
0.05	85	t(29)=-8.41, p<0.0001	t(29)=-5.17, p=0.0002	t(29)=20.66, p<0.0001
0.05	90	t(29)=-8.19, p<0.0001	t(29)=-4.39, p=0.0001	t(29)=18.31, p<0.0001
0.05	95	t(29)=-8.04, p<0.0001	**t(29)=-3.10, p=0.004**	t(29)=15.06, p<0.0001
0.005	85	t(29)=-8.42, p<0.0001	t(29)=-5.17, p=0.0002	t(29)=20.66, p<0.0001
0.005	90	t(29)=-8.19, p<0.0001	t(29)=-4.39, p=0.0001	t(29)=18.31, p<0.0001
0.005	95	t(29)=-8.04, p<0.0001	**t(29)=-3.10, p=0.004**	t(29)=15.06, p<0.0001
0.0005	85	t(29)=-8.42, p<0.0001	t(29)=-5.17, p=0.0002	t(29)=20.66, p<0.0001
0.0005	90	t(29)=-8.19, p<0.0001	t(29)=-4.39, p=0.0001	t(29)=18.31, p<0.0001
0.0005	95	t(29)=-8.04, p<0.0001	**t(29)=-3.10, p=0.004**	t(29)=15.06, p<0.0001
0.000 05	85	t(29)=-8.42, p<0.0001	t(29)=-5.17, p=0.0002	t(29)=20.66, p<0.0001
0.000 05	90	t(29)=-8.19, p<0.0001	t(29)=-4.39, p=0.0001	t(29)=18.31, p<0.0001
0.000 05	95	t(29)=-8.04, p<0.0001	**t(29)=-3.10, p=0.004**	t(29)=15.05, p<0.0001

Table S6: Results of a two-sided t-test of the difference between the subject-level F1-scores for the tool stimulus class of each of the competing analysis approaches with the subject-level F1-scores of DeepLight. The t-test comparison was repeated for each combination of percentile- and P-threshold (for details on the F1-score comparison procedure, see the Results section of the main text and Supplementary Information section 3). T-tests were performed using a Bonferroni adjusted alpha level of 0.0014 (0.05/36). Bold font indicates t-tests with p-values greater than 0.0014.

References

[Abraham et al., 2014] Abraham, A., Pedregosa, F., Eickenberg, M., Gervais, P., Mueller, A., Kossaifi, J., Gramfort, A., Thirion, B., and Varoquaux, G. (2014). Machine learning for neuroimaging with scikit-learn. *Frontiers in Neuroinformatics*, 8:14.

[Benjamini and Hochberg, 1995] Benjamini, Y. and Hochberg, Y. (1995). Controlling the false discovery rate: a practical and powerful approach to multiple testing. *Journal of the Royal statistical society: series B (Methodological)*, 57(1):289–300.

[Genovese et al., 2002] Genovese, C. R., Lazar, N. A., and Nichols, T. (2002). Thresholding of statistical maps in functional neuroimaging using the false discovery rate. *Neuroimage*, 15(4):870–878.

[Gorgolewski et al., 2011] Gorgolewski, K., Burns, C. D., Madison, C., Clark, D., Halchenko, Y. O., Waskom, M. L., and Ghosh, S. S. (2011). Nipype: a flexible, lightweight and extensible neuroimaging data processing framework in python. *Frontiers in Neuroinformatics*, 5:13.

[Gramfort et al., 2013] Gramfort, A., Thirion, B., and Varoquaux, G. (2013). Identifying predictive regions from fmri with tv-l1 prior. In *Pattern Recognition in Neuroimaging (PRNI), 2013 International Workshop on*, pages 17–20. IEEE.

[Helfinstein et al., 2014] Helfinstein, S. M., Schonberg, T., Congdon, E., Karlsgodt, K. H., Mumford, J. A., Sabb, F. W., Cannon, T. D., London, E. D., Bilder, R. M., and Poldrack, R. A. (2014). Predicting risky choices from brain activity patterns. *Proceedings of the National Academy of Sciences of the United States of America*, 111(7):2470–2475.

[Holmes and Friston, 1998] Holmes, A. and Friston, K. (1998). Generalisability, random effects & population inference. *NeuroImage*, 7:S754.

[Kiefer et al., 1952] Kiefer, J., Wolfowitz, J., et al. (1952). Stochastic estimation of the maximum of a regression function. *The Annals of Mathematical Statistics*, 23(3):462–466.

[Lindquist et al., 2009] Lindquist, M. A., Loh, J. M., Atlas, L. Y., and Wager, T. D. (2009). Modeling the hemodynamic response function in fmri: efficiency, bias and mis-modeling. *Neuroimage*, 45(1):S187–S198.

[Reverberi et al., 2018] Reverberi, C., Kuhlen, A. K., Seyed-Allaei, S., Greulich, R. S., Costa, A., Abutalebi, J., and Haynes, J.-D. (2018). The neural basis of free language choice in bilingual speakers: Disentangling language choice and language execution. *NeuroImage*, 177:108–116.

[Schuck et al., 2016] Schuck, N. W., Cai, M. B., Wilson, R. C., and Niv, Y. (2016). Human orbitofrontal cortex represents a cognitive map of state space. *Neuron*, 91(6):1402–1412.

[Weygandt et al., 2012] Weygandt, M., Schaefer, A., Schienle, A., and Haynes, J.-D. (2012). Diagnosing different binge-eating disorders based on reward-related brain activation patterns. *Human brain mapping*, 33(9):2135–2146.

[Yarkoni et al., 2011] Yarkoni, T., Poldrack, R. A., Nichols, T. E., Van Essen, D. C., and Wager, T. D. (2011). Large-scale automated synthesis of human functional neuroimaging data. *Nature Methods*, 8(8):665.
